# From the Surface to the Deep-Sea: Bacterial Distributions across Polymetallic Nodule Fields in the Clarion-Clipperton Zone of the Pacific Ocean

**DOI:** 10.3389/fmicb.2017.01696

**Published:** 2017-09-08

**Authors:** Markus V. Lindh, Brianne M. Maillot, Christine N. Shulse, Andrew J. Gooday, Diva J. Amon, Craig R. Smith, Matthew J. Church

**Affiliations:** ^1^Daniel K. Inouye Center for Microbial Oceanography: Research and Education, University of Hawai‘i at Mānoa Honolulu, HI, United States; ^2^National Oceanography Centre, University of Southampton Waterfront Campus Southampton, United Kingdom; ^3^Department of Oceanography, University of Hawai‘i at Mānoa Honolulu, HI, United States

**Keywords:** bacterial diversity, population dynamics, biogeography, deep-sea mining, polymetallic nodules, colonization, export, Clarion-Clipperton Zone

## Abstract

Marine bacteria regulate fluxes of matter and energy essential for pelagic and benthic organisms and may also be involved in the formation and maintenance of commercially valuable abyssal polymetallic nodules. Future mining of these nodule fields is predicted to have substantial effects on biodiversity and physicochemical conditions in mined areas. Yet, the identity and distributions of bacterial populations in deep-sea sediments and associated polymetallic nodules has received relatively little attention. We examined bacterial communities using high-throughput sequencing of bacterial 16S rRNA gene fragments from samples collected in the water column, sediment, and polymetallic nodules in the Pacific Ocean (bottom depth ≥4,000 m) in the eastern Clarion-Clipperton Zone. Operational taxonomic units (OTUs; defined at 99% 16S rRNA gene identity) affiliated with JTB255 (Gammaproteobacteria) and *Rhodospirillaceae* (Alphaproteobacteria) had higher relative abundances in the nodule and sediment habitats compared to the water column. *Rhodobiaceae* family and *Vibrio* OTUs had higher relative abundance in nodule samples, but were less abundant in sediment and water column samples. Bacterial communities in sediments and associated with nodules were generally similar; however, 5,861 and 6,827 OTUs found in the water column were retrieved from sediment and nodule habitats, respectively. Cyanobacterial OTUs clustering among *Prochlorococcus* and *Synechococcus* were detected in both sediments and nodules, with greater representation among nodule samples. Such results suggest that vertical export of typically abundant photic-zone microbes may be an important process in delivery of water column microorganisms to abyssal habitats, potentially influencing the structure and function of communities in polymetallic nodule fields.

## Introduction

Polymetallic nodules containing high levels of manganese, iron, cobalt, and nickel can form extensive fields on abyssal plains, raising the prospect of future deep-sea mining on a commercial scale (Ghosh and Mukhopadhyay, 2000; Wegorzewski and Kuhn, 2014). There is a growing body of research on marine organisms, including bacteria, associated with polymetallic nodules (see e.g., Wu et al., 2013; Gooday et al., 2015; Amon et al., 2016; Dahlgren et al., 2016; Glover et al., 2016; Shulse et al., 2016; Vanreusel et al., 2016). Other studies have sought to understand potential environmental effects of mining waste tailings released back into the water column following planned deep-sea mining operations (Oebius et al., 2001; Rolinski et al., 2001). In the abyssal Pacific Ocean, work focused on microbial diversity identified specific bacterial operational taxonomic units (OTUs) associated with nodules, including those clustering among Alpha- and Gammaproteobacteria (Tully and Heidelberg, 2013; Wu et al., 2013; Blöthe et al., 2015; Shulse et al., 2016). Moreover, it has been suggested that particular bacterial populations have notable mechanisms of energy cycling (Tully and Heidelberg, 2016), and are involved in the precipitation of metals potentially drive the formation and maintenance of nodules (Burnett and Nealson, 1981; Myers and Nealson, 1988; Ehrlich, 2001; Wang et al., 2008; Wu et al., 2013; Blöthe et al., 2015). However, to date, there is still relatively little known about the identity and distributions of bacteria dwelling in deep-sea sediments and associated with polymetallic nodules.

Despite being linked by sinking particles, there appear to be significant differences between pelagic and abyssobenthic bacterial communities, with only a small proportion of OTUs seemingly shared between these habitats (Zinger et al., 2011). Benthic habitats often harbor greater bacterial diversity due in large part to increased relative abundances of rare OTUs (Zinger et al., 2011; Shulse et al., 2016). Particles, including bacterial cells, originating from the upper ocean, however, influence the quality and quantity of food reaching the deep-sea benthos and therefore energy flow and ecosystem functioning within the benthic realm (Smith et al., 2001, 2008; Leon et al., 2002; Azam and Malfatti, 2007). Picoplanktonic bacteria, such as cyanobacteria, can be exported from the upper ocean to the deep sea in association with phytodetritus, a term used to describe phytoplankton aggregates that gravitationally settle from the photic zone to the seafloor relatively undegraded (Billett et al., 1983; Thiel et al., 1990; Smith et al., 1996; Richardson and Jackson, 2007; Tang et al., 2014). Studies indicate that the flux of phytodetritus to the benthos in the Pacific nodule fields appears to be relatively low (Gardner et al., 1984; Gooday et al., 1992; Smith et al., 1996, 2008; Beaulieu, 2002; Lutz et al., 2007; Wedding et al., 2013) but can have a disproportionately large influence on benthic organisms; influencing distributions, feeding strategies, reproductive patterns, and the diversity of deposit-feeding taxa (Smith et al., 1996, 2008; Smith and Demopoulos, 2003; Wigham et al., 2003).

We examined the structure of bacterial communities in water-column, sediment and polymetallic-nodule habitats at water depths ≥4,000 m in the eastern Clarion-Clipperton Zone (CCZ; Pacific Ocean) through amplification and sequencing of bacterial 16S rRNA genes collected in the in the CCZ of the eastern Pacific Ocean. The main focus in the present study was to characterize bacterial community structure in this region to highlight key similarities and differences in the types of bacteria retrieved from the distinct habitats sampled. In addition, we compared the bacterial communities observed in potential mining areas (exploration contract areas) to a location in an Area of Particular Environmental Interest (APEI) in the eastern CCZ protected from mining by the International Seabed Authority. The resulting analyses revealed bacterial OTUs specifically associated with nodules that are typically observed in the upper ocean. These findings suggested that such microorganisms contribute to sinking particle flux and may play a role in catalyzing or sustaining nodule formation.

## Materials and methods

### Field sampling

**Figure 1:**
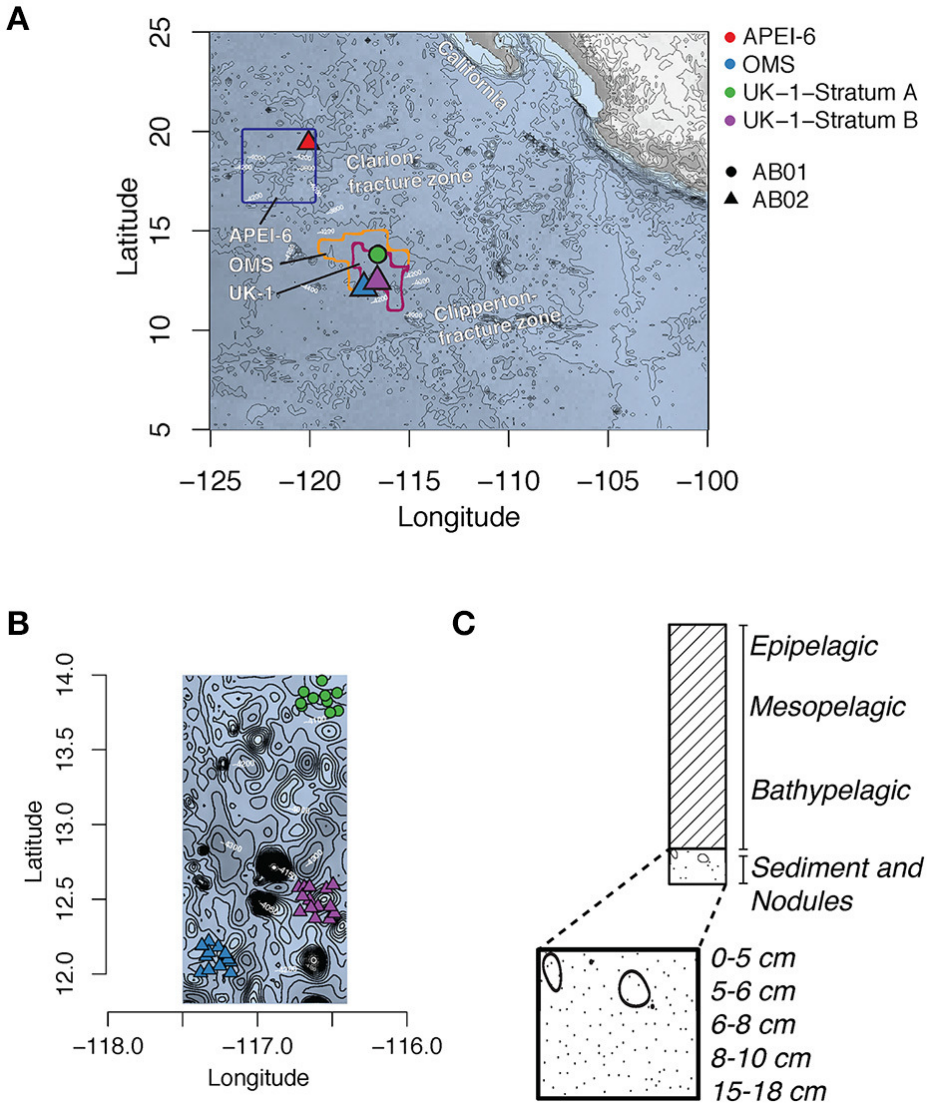
Map of the Clarion-Clipperton Zone **(A)** with map showing the location and bathymetry of UK-1 Stratum A (AB01 cruise), UK-1 Stratum B and OMS Stratum A (AB02 cruise) **(B)**, and the distribution of water column, sediment and nodule collection of samples **(C)**. Contours in A and B denote bottom depth.

Seawater sediment samples, and polymetallic nodules were collected during the ABYSSLINE research cruise AB02 (R/V *Thomas G Thompson* cruise TN319, February–March 2015; hereafter AB02) in the eastern CCZ. Samples were obtained within two areas licensed for polymetallic-nodule exploration by the International Seabed Authority (ISA), the body that regulates activities on the seafloor in areas beyond national jurisdiction. One license area (UK-1; centered ~at 13° 49′ N, 116° 36′ W) is licensed to UK Seabed Resources Ltd and the other (OMS; centered ~at 13° 49′ N, 116° 36′ W) to Ocean Mineral Singapore (OMS; Figure 1). Inside each exploration contract area, samples were taken at 12 randomly chosen stations within a 30 × 30 km stratum (Stratum B in UK-1 and Stratum A in OMS). Additional samples were collected within a 30 × 30 km study stratum from the northeastern corner of the 400 × 400 km APEI region 6, centered at 19° 30.0 N, 120° 8.6 W. The sampling scheme is summarized in Table S1. We also included selected samples obtained during an earlier sampling opportunity (cruise AB01 aboard the R/V *Melville*, October 2013) from a second 30 × 30 km stratum (UK-1 Stratum A; Shulse et al., 2016).

Seawater samples were collected from eight discrete depths within the water column (5, 125, 300, 500, 1,000, 2,000, 3,000 m), and at 5 m altitude above the seafloor at bottom depths ranging from 4,025 to 4,218 m, using a conductivity, temperature, depth (CTD) rosette equipped with 10 L Niskin sampling bottles. The CTD sensors included dual SBE3 temperature, SBE4 conductivity and SBE43 oxygen sensors, and a fluorometer (Wetlabs ECO-FLTRD). Samples for subsequent extraction of DNA were subsampled from the rosette bottles into acid-washed, Milli-Q rinsed, polycarbonate bottles. Planktonic biomass was harvested by filtering 2, 4, and 10 L of seawater from 5, 150–500, and >500 m depths, respectively, onto 25 mm diameter, 0.2 μm pore size Supor filters (Pall Corporation). Filters were placed in 2 mL microcentrifuge tubes containing lysis buffer AP1 (Qiagen) and glass beads (0.1 and 0.5 mm, Biospec products), flash frozen in liquid nitrogen, and stored at −80°C until extraction.

Nodules and sediments were aseptically sampled from either a box core (nodules) or a megacore (nodules and sediments). Sub-sections of sediments from each of five sediment depth layers (0–5, 5–6, 6–8, 8–10, 15–18 cm) were obtained by shipboard subcoring of box cores or megacores using sterile 20 mL syringes. Sediments extruded from sub-cores were flash frozen in liquid nitrogen and stored in sterile Whirl-Pak bags (Nasco, Fort Atkinson, Wisconsin) at −80°C. Nodules were gently rinsed with 0.2 μm-filtered near-bottom waters to remove sediment adhering to their surfaces before being flash frozen in liquid nitrogen and stored in sterile Whirl-Pak bags at −80°C. Contamination of nodule and sediment samples by seawater from the overlying water column could have occurred during sampling. Although a few nodule samples were collected with a boxcorer, the majority of nodule samples analyzed in the current study were collected using a megacorer. This method of sampling completely seals the bottom of the core upon firing at the seafloor, making contamination from surface waters unlikely. In addition, we tried to minimize contamination issues by rinsing nodules with 0.2 μm filtered near-bottom water shipboard, and prior to DNA extraction, nodules were rinsed again with autoclaved 0.2 μm filtered near-bottom waters.

### DNA extraction, PCR, sequence processing, and analysis

DNA was extracted as described in Shulse et al. (2016). Briefly, DNA was obtained from planktonic biomass using the DNeasy Plant MiniKit (Qiagen), following a protocol slightly modified from the manufacturer's suggestions, including the addition of Proteinase K and bead-beating for additional cell disruption prior to the extraction (Paerl et al., 2008). Extracted DNA was stored at −80°C. Under sterile laboratory conditions, nodules were rinsed with 0.2 μm-filtered, autoclaved bottom (~4,000 m) seawater, and broken using an autoclaved mortar and pestle. DNA was extracted from a ~500 mg piece of each nodule. Extraction of DNA from nodules and sediments was performed using the FastDNA Spin Kit for Soil (MP Biomedicals, USA) following the manufacturer's protocol, modified as follows: homogenization was performed in a Mini-Beadbeater-16 (Biospec Products, Bartlesville, Oklahoma) and centrifugation following homogenization was extended to 15 min. An extraction blank (FastDNA Spin Kit for Soil spin column with no sample added) was processed alongside the samples. DNA concentrations from both nodules and seawater samples were determined using the Qubit 2.0 Fluorometer and the Qubit dsDNA High Sensitivity Assay kit (Life Technologies).

For amplicon processing the primers used in the present paper differed from those used by Shulse et al. (2016) and some of the samples included in the study performed by Shulse et al. (2016) were here re-amplified and sequenced with our primers. Overall, this study greatly expanded the spatial region of the CCZ including new areas compared to those included in Shulse et al. (2016). Thus, amplicon processing for all samples collected during AB02, as well as selected samples within the UK-1 Stratum A sampled during AB01 (Shulse et al., 2016), was performed as described in Lindh et al. (2015). We targeted the bacterial 16S rRNA gene to keep focus on one domain and avoid primer bias resulting from targeting multiple domains, i.e., archaea, bacteria and eukaryotes. Briefly, bacterial 16S rRNA genes were amplified using primers 341F and 805R (Herlemann et al., 2011) following the nested polymerase chain reaction (PCR) protocol of Hugerth et al. (2014) with some modifications. Specifically, amplification reactions were carried out in duplicate for each sample and an annealing temperature of 65°C was utilized for the initial round of PCR. The resulting amplicons were purified by spin-column centrifugation using E.Z.N.A.® Cycle Pure kit (Omega Biotek), and sequenced on an Illumina Miseq (Illumina, USA) platform at the Hawai'i Institute for Marine Biology (HIMB), University of Hawaii, USA using the 300 bp paired-end setting. Raw sequence data generated from Illumina Miseq were processed using the UPARSE pipeline (Edgar, 2013). After quality filtering and removal of plastid and archaeal sequences and after subtracting all hits of OTUs found in the blank filter control, a total of 4.3 million bacterial sequences were utilized for subsequent analyses. Taxonomy was determined against the SINA/SILVA database (SILVA123; Quast et al., 2013). The final operational taxonomic unit (OTU) table consisted of 447 samples with 46,661 OTUs delineated at 99% 16S rRNA gene identity with an average of 9694.7 ± 892.7 sequences per sample. We normalized the OTU tables by dividing the number of sequences for each OTU by the total number of sequences in each sample, i.e., normalization to relative abundances. For alpha diversity measures, the total OTU table was subsampled to 5,000 sequences per sample. DNA sequences have been deposited in the National Center for Biotechnology Information (NCBI) Sequence Read Archive under accession numbers SRP078393, SRP078394, SRP078395, SRP078397, and SRP078396.

### Statistical analyses and graphical outputs

Seawater samples were divided into vertical zones corresponding to the epipelagic zone (0–200 m), mesopelagic zone (200–1,000 m), and bathy- to abyssopelagic zone (1,000–3,000 m), and abyssopelagic (3,000 m—seafloor). All statistical tests were performed in R 3.3.1 (R Development Core Team, 2014) using the package “Vegan” (Oksanen et al., 2010). Graphical outputs were made in R 3.3.1 using the package “ggplot2” (Wickham, 2009).

## Results

### Bacterial diversity associated with sediments, nodules, and seawater

Alpha (number of OTUs per sample and Shannon diversity index) and beta diversity (Chao-1 index) metrics were lower in the water column compared to nodules and sediments. Diversity increased with depth in the water column, with the lowest mean value for all three diversity metrics across all sample types observed in the epipelagic zone (Figure S1A). Nodule samples displayed higher alpha and beta diversity than seawater samples but lower diversity than sediment samples at all sediment layers except 15–18 cm. The highest overall Shannon diversity index (7.88) was observed in the 0–5 cm sediment layer. For each sediment layer and for nodule samples, there were significant differences in the Shannon index between the four study areas (Two-way ANOVA, *p* < 0.001, df = 16, *n* = 331), with the APEI-6 location having lower diversity overall compared to OMS Stratum A, UK-1 Stratum A and UK-1 Stratum B (Tukey's *post-hoc* test, *p* < 0.01, df = 3). Shannon diversity indices in UK-1 Stratum B and OMS Stratum A were also significantly different (Tukey's *post-hoc* test, *p* < 0.01, df = 1). Notably, mean Shannon diversity for nodule samples, although not for water column and sediment samples, was lower in the APEI-6 location than in the claim-area strata (Figure S1B).

**Figure 2:**
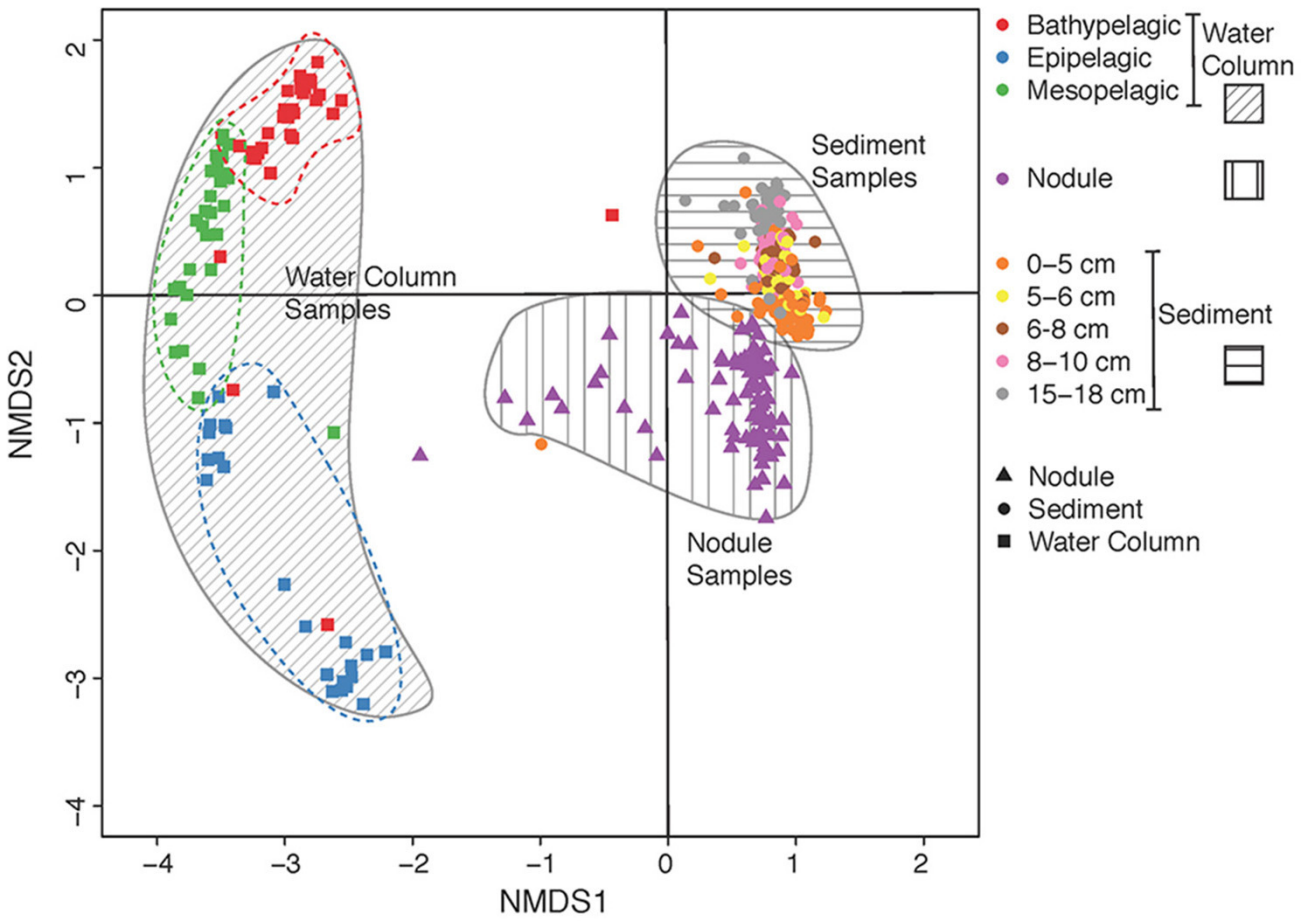
Non-metric multidimensional scaling (nMDS) plot of microbial community dissimilarity (beta diversity) calculated from Bray-Curtis distances. Gray areas denote visual clustering of samples into different habitat (water column, sediment and nodules) and dashed lines denote visual clustering of vertical segments within the water column (i.e., the epipelagic, mesopelagic, and bathypelagic zones).

Differences in community composition between the water column zones, sediment layers, nodule samples and study areas were examined using Bray-Curtis distance estimation (Figure 2). Water-column communities clustered separately and were significantly different from both sediment and nodule communities, and sediment communities were significantly different from nodule communities (Figure 2; PERMANOVA, *p* < 0.01, df = 3, *n* = 447). In addition, community composition was significantly different between contract areas and APEI-6 within each of the water column zones, sediment layers, and nodule samples (PERMANOVA, *p* < 0.01, df = 8, *n* = 447). Overall, there was considerable variation in bacterial community composition among the water-column samples. Moreover, near-bottom water (>4,000 m) bacterial communities were significantly different from those associated with sediments and nodules, regardless of the area sampled (PERMANOVA, *p* < 0.01, df = 6, *n* = 374).

### Community structure

**Figure 3:**
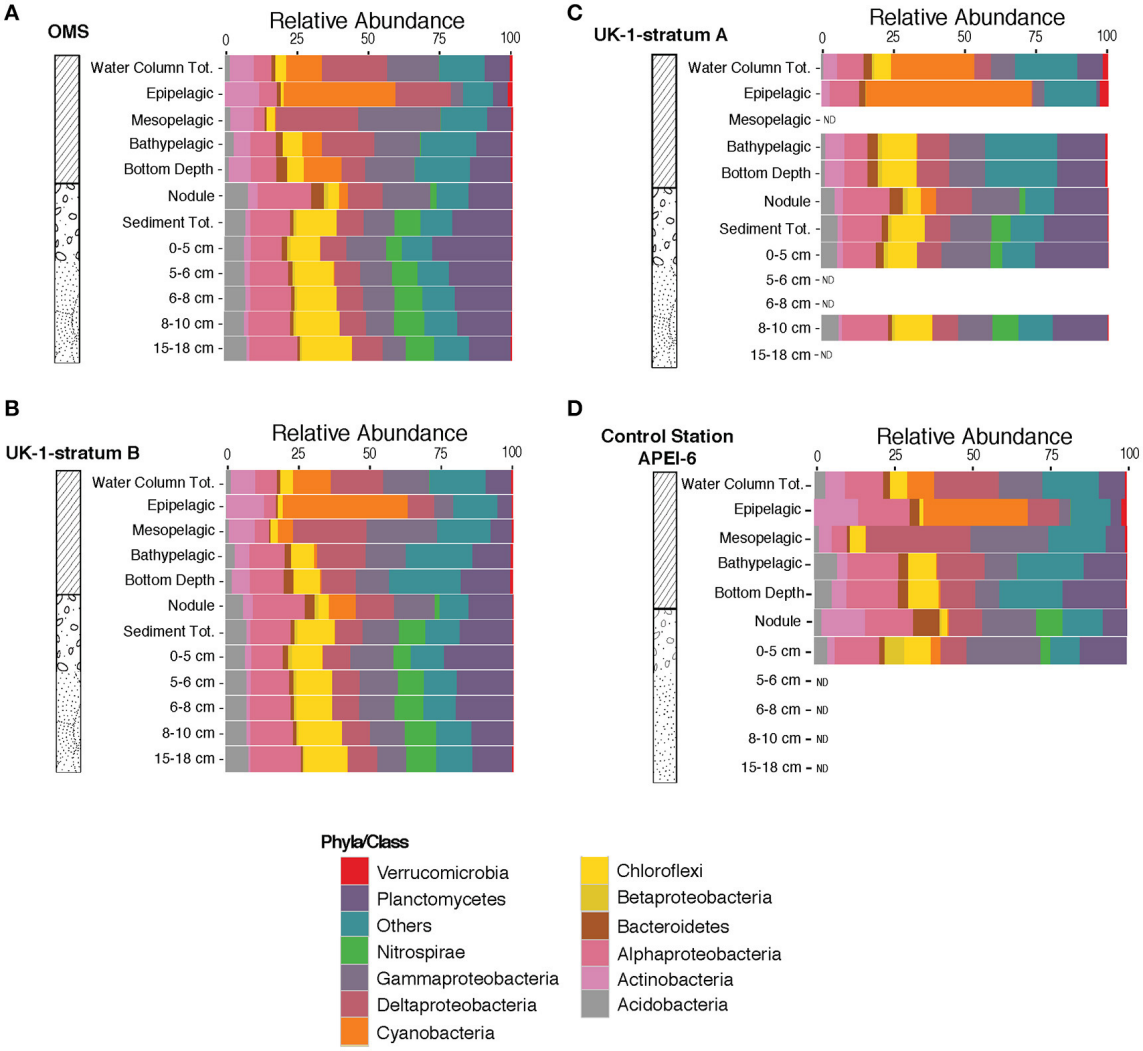
Relative abundances of operational taxonomic units binned at phyla/class level in the three different strata OMS Stratum A **(A)**, UK-1 Stratum B **(B)**, UK-1 Stratum A **(C)**, and APEI-6 **(D)**. Relative abundances were averaged over all samples collected in each stratum and different habitat (water column, sediment and nodules) and vertical segments within habitats (i.e., the epipelagic, mesopelagic, and bathypelagic zones, the 0–5, 5–6, 6–8, 8–10, and 15–18 cm sediment layers and nodules).

We investigated the bacterial taxonomic composition at phylum/class level in order to establish the distribution of taxa in the different sampled habitats. Bacterial taxonomic composition was distinct within the various vertical zones of the water column. Cyanobacterial OTUs were dominant in the epipelagic zone, while OTUs from members of the Acidobacteria were nearly absent in this region of the upper ocean (Figure 3). OTUs belonging to Nitrospirae were represented in all sediment layers and nodule samples, but in very low relative abundances throughout the water column. Interestingly, cyanobacterial OTUs were present in nodule samples, but virtually absent from the sediment layers (Figure 3). The protected area (APEI-6) had a similar overall distribution of taxa at this phylum/class level (Figure 3D); however, the nodule samples from the APEI-6 region did not contain cyanobacterial OTUs, although these OTUs were present in the 0–5 cm sediment layers from the APEI study site.

### Vertical distribution and habitat specialization among individual OTUs

**Figure 4:**
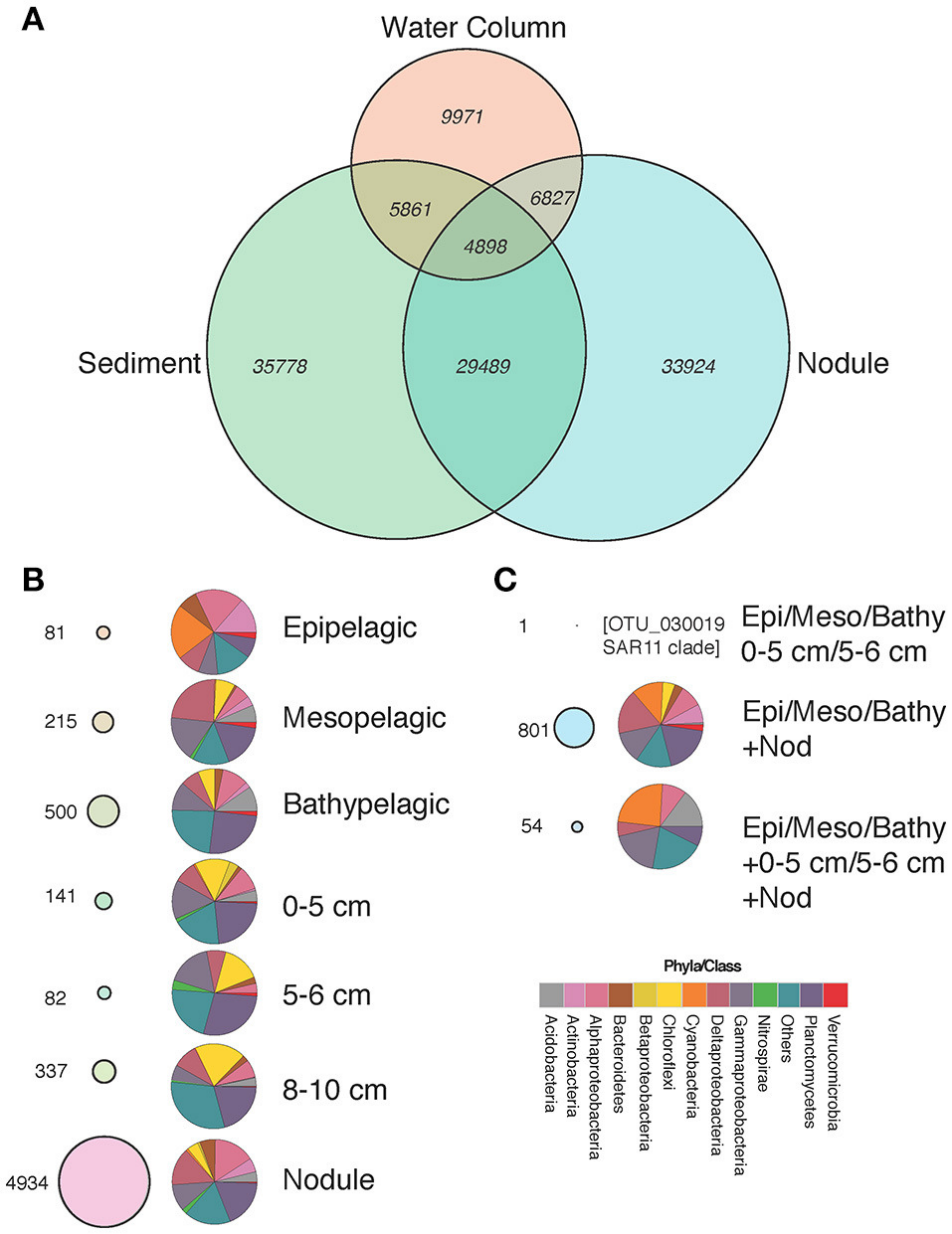
Venn diagram showing the distribution of OTUs and the number of shared OTUs between habitats **(A)**, “Specialist” OTUs and their taxonomic affiliation **(B)**, and “moderate generalist” and “generalist” OTUs and their taxonomic affiliation **(C)**. All bubbles of “specialist,” “moderate generalists” and “generalists” in **(A–C)** are proportional to each other.

We investigated the distribution of individual OTUs and their taxonomic affiliation in the various habitats, i.e. water-column zones, sediment layers, and nodules (Figure 4). The total number of OTUs detected within the water column, sediment, and nodules were 9,971, 35,778, and 33,924, respectively (Figure 4A). The water column shared 5,861 and 6,827 OTUs with the sediment and nodule habitats, respectively. Most (*n* = 29,489) of the retrieved OTUs were shared between the sediment and nodule habitats (Figure 4A), with a total of 4,898 OTUs present in all three habitats.

We further classified OTUs into three major groups according to their presence/absence patterns in the different water-column zones, sediment layers, and nodules, and between the three habitats sampled (nodules, seawater, and sediments). We classified these OTUs as: (i) “specialists,” those present only in one vertical region within each habitat (Figure 4B, Table S2); (ii) “generalists,” present in all habitats and individual vertical regions (Figure 4C, Table S2); and (iii) “moderate generalists,” those present in at least two habitats and in one or more vertical regions of each habitat (Figure 4C, Table S2). In the water column, the bathypelagic samples (>1,000 m) contained the highest number of “specialist” OTUs (*n* = 500), followed by the mesopelagic (300–1,000 m; *n* = 215) and epipelagic (0–300 m; *n* = 81), contributing to 0.02, 0.06, and 0.27% of total sequences, respectively. Sediment “specialists” were most common in the 8–10 cm layer (*n* = 337) followed by the 0–5 cm (*n* = 141) and the 5–6 cm (*n* = 82) layers, contributing to 0.07, 0.015, and 0.005% of the total OTUs, respectively. No “specialist” OTUs were found in the 6–8 or 15–18 cm layers. Specialist OTUs detected only in sediment samples included relatively high numbers of Planctomycetes, Chloroflexi and the group “Others” (consisting of all other phyla/classes not named in the current analysis) that together made up 25–50% of all OTUs present in those layers (Figure 4, Table S2). To examine the depth distributions of Chloroflexi and Planctomycetes populations, we plotted the relative abundances of these groups in the different sediment layers (Figure S2). Both groups showed approximately equal distributions across all sediment layers, although there tended to be more Chloroflexi OTUs in higher relative abundances in the 15–18 cm layer than in other layers (Figure S2). Compared to the other habitats, nodules had the highest number of specialist OTUs (*n* = 4,934), representing 5% of the total number of sequences. The top 20 OTUs with the widest distributions among nodule specialists (Table S3) occurred at >90% of all sampled sites and included those assigned to the Alphaproteobacterial *Rhodobiaceae* (OTU_1278, OTU_355, OTU_121, and OTU_604), and Gammaproteobacterial JTB255 marine benthic group (OTU_50, OTU_208, OTU_145, OTU_2609, and OTU_764), and *Vibrio* (OTU_185, OTU_33, OTU_131, OTU_222, OTU_258, and OTU_719).

No OTUs were found across all water column strata, nodule habitats and sediment layers, but 54 OTUs were shared among the≤ 6 cm sediment layers, nodules, and the epipelagic, mesopelagic, and bathypelagic regions of the water column (i.e., they were only absent from the 6–8, 8–10, and 15–18 cm sediment layers). These “generalists” OTUs corresponded to 1% of all sequences (Table S2), and included approximately equal proportions of cyanobacteria, gammaproteobacteria, and the group “Others,” with planctomycetes, acidobacteria, deltaproteobacteria, and alphaproteobacteria occurring at lower relative abundances. Among the most abundant “generalist” OTUs were those closely related to the cyanobacteria *Prochlorococcus* spp. (OTU_77, OTU_206, OTU_301, and OTU_2134) and *Synechococcus* spp. (OTU_176, OTU_267, and OTU_308), OTUs belonging to the SAR324 clade of the Deltaproteobacteria (OTU_318), and OTUs most similar to *Candidatus* Actinomarina (OTU_1085 and OTU_1725). We also evaluated which of these specific OTUs had the widest distributions in the study region (Table S4). Among the top 20 OTUs with the widest distribution, three groupings were evident: (i) those clustering among the JTB255 marine benthic Gammaproteobacteria (OTU_2, OTU_18, OTU_29, OTU_106, OTU_31, OTU_46, OTU_56, OTU_74, OTU_67); (ii) those belonging to the *Rhodospirillaceae* in the Alphaproteobacteria (OTU_4, OTU_27, OTU_34, OTU_24, OTU_170, OTU_84, OTU_51); (iii) those belonging to Subgroup 21 of the Acidobacteria (OTU_16, OTU_66, OTU_94, OTU_92). No OTUs were found in high relative abundances in all three habitats (seawater, sediments, and nodules), although some OTUs occupied >85% of all sampled habitats and vertical segments.

The number of “moderate generalists” shared among the water column and sediment habitats were low, typically < 10 OTUs, and contributed very little to the total number of sequences. Although, the sediment and nodule habitats shared most OTUs (Figure 4C), no OTUs were shared between sediment layers ≥6 cm and nodules. There were 1,759 OTUs shared between the 0–5 cm sediment layer and the nodules and 1,757 shared between the 0–5 and 5–6 cm sediment layers and the nodules.

“Moderate generalist” OTUs (*n* = 801) detected in all water column zones (epipelagic, mesopelagic, and bathypelagic) and in nodule samples corresponded to 2.5% of total sequences (Figure 4, Table S2). They comprised approximately equal proportions of alphaproteobacteria, cyanobacteria, deltaprotebacteria, gammaproteobacteria, nitrospirae, and the group “Others.” Conversely, members of the planctomycetes, verrucomicrobia, acidobacteria, bacteroidetes, and betaproteobacteria were less well-represented among the water column/nodule “moderate generalist” OTUs. The “moderate generalists” OTUs (*n* = 163) that were only found in the epipelagic region and nodule samples represented 0.07% of all sequences. Among the OTUs found in both water column and nodule samples, but not in sediment samples, some were closely related to typically abundant ocean surface taxa, such as *Prochlorococcus* (Table S2). These included OTU_213, OTU_694, and OTU_438, which were closely related (>99% 16S rRNA gene identity) to *Prochlorococcus marinus* str. MIT 9303 (Kettler et al., 2007).

**Figure 5:**
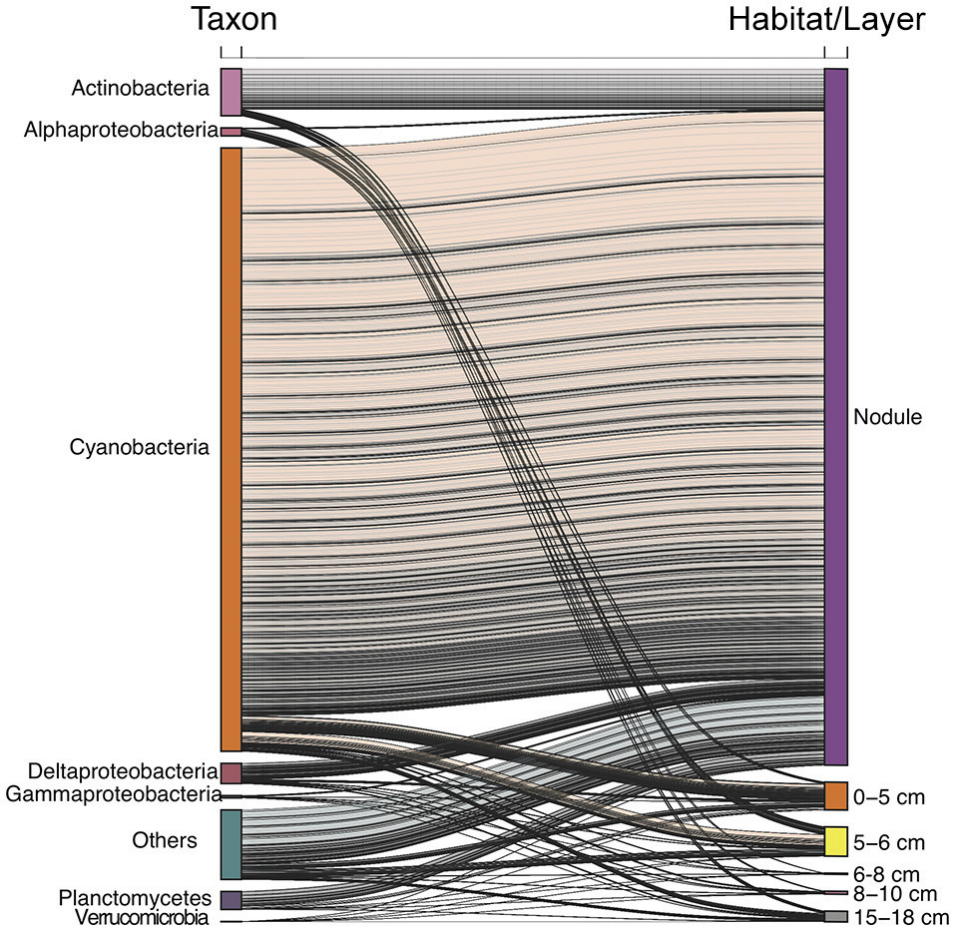
Alluvial plot of abundant (>1% relative abundance) epipelagic OTUs and their distribution in different sediment layers and the nodule habitat.

Interestingly, OTUs that were abundant in the nodule habitat were, in general, also relatively abundant in water column and sediment samples (Figure S3). In particular, there were significant relationships between the relative abundances of OTUs in nodule and water column samples (least squares linear regression, *R*^2^ ≥ 0.63, *p* < 0.01; Figure S3A). The relative abundances of nodule OTUs were also correlated with the relative abundance of sediment OTUs, except for the 6–8 and 8–10 cm sediment layers (Figure S3B). Notably, most of the abundant epipelagic OTUs also found in the nodule habitat were those related to cyanobacteria (Figure 5). Although the majority (~75%) of sequences related to cyanobacteria occurred in the epipelagic zone, ~10% were retrieved from nodules compared to <1% from sediments (Figure S4). Among the most abundant OTUs detected in this study (Figure S5), some assigned to the cyanobacteria were relatively abundant (>1%, Figure S5) in both the epipelagic and nodule habitats. In contrast, most OTUs that were abundant in the sediment layers were confined to this habitat.

We also sought to place the observed pattern of selectively preserved water column OTUs in nodules in context with available microbial and eukaryotic sequence data obtained from the same geographical area during the AB01 cruise (Shulse et al., 2016). We performed network analyses to evaluate the frequency of co-occurrence of eukaryotic 18S rRNA and bacterial 16S rRNA OTUs. The network analyses revealed highly significant correlations between the occurrences of particular foraminiferal and bacterial OTUs (Figure S6). Among others, two cyanobacterial OTUs (including those assigned to *Prochlorococcus* and *Synechococcus*) formed significant co-occurrences with foraminiferal OTUs. All foraminiferal OTUs identified in the network analysis (except for the agglutinated *Trochammina* sp.) clustered among shallow-water calcareous species, the majority of them clustering among so-called “larger foraminifera” which are best described in shallow-water reef environments.

## Discussion

In the present paper we investigated bacterial community structure and distribution collected from the AB01 (Shulse et al., 2016) and AB02 cruises in the Clarion-Clipperton Zone (CCZ) of the eastern Pacific Ocean in seawater samples ranging from the surface to the deep-sea (>4,000 m), including sediment and polymetallic nodules. We included samples obtained by Shulse et al. (2016) but here re-analyzed using our primers focusing on bacterial 16S rDNA. Therefore, by sampling new areas compared to those included in Shulse et al. (2016) but also including samples from that study we greatly expanded our understanding of bacterial distributions in the commercially valuable CCZ area.

Our results are in general agreement with previous evidence that microbial alpha and beta diversity in marine sediments is greater than in other aquatic environments (Lozupone and Knight, 2007), and that while alpha diversity is higher associated with nodules than in seawater, nodule communities are less diverse than sediment communities (Wu et al., 2013; Zhang et al., 2014; Shulse et al., 2016). In addition, the location sampled in the protected area, APEI-6, had lower alpha and beta diversity compared to strata within the exploration contract areas, suggesting that northeast corner of the APEI-6 has a different bacterial community from those in the UK-1 and OMS claim areas ~1,200 km away. However, Shannon diversity for nodules collected in APEI-6 fall within the range of that observed for nodules from OMS Stratum A and UK-1 stratum B (Figure S1B). Comparing our results from the APEI sampling to UK-1 and OMS regions is limited by differences in sample sizes: only one water column sample and two box core samples were collected in the northeast corner of APEI-6, while a much larger number of samples were collected in the UK-1 and OMS claim areas. Moreover, because we sampled only the northeast corner of a vast, 160,000 km^2^ area of the APEI, our sampled location may not be representative of the APEI-6 region. Additional studies will be needed to determine how well the bacterial assemblages in the ISA's nine APEIs represent the bacterial communities in the 15 mining claim areas spanning the CCZ (Figure 1).

Previous studies have shown that vertical changes in pressure, temperature, and salinity can be drivers of diversity and compositional shifts among microbial assemblages (Pommier et al., 2010; Zinger et al., 2011; Sunagawa et al., 2015; Salazar et al., 2016). The composition of both sediment and nodule assemblages appeared more similar than assemblages sampled between the vertically distinct regions of the water column. In agreement, Shulse et al. (2016) found similar distributions in a smaller area of the CCZ. Differences in community composition likely reflect vertical differences in physiochemical conditions and ultimately energy supply. Intriguingly, near-bottom-water (>4,000 m) bacterial communities were distinct from those associated with sediments and nodules, regardless of sampling area. These findings extend a previous notion, based on a single sampling region, on the same type distinction between near-bottom waters to sediment and nodule samples (Shulse et al., 2016) to now encompass three different sampling areas within the CCZ. In addition, alpha and beta diversity were higher in the nodule and sediment samples compared to diversity in the near-bottom waters. Together, these results suggest the sediment and nodule habitats may offer a broader niche range and greater probability of speciation than the water column.

Consistent with previous studies (Tully and Heidelberg, 2013; Wu et al., 2013; Blöthe et al., 2015; Shulse et al., 2016), we found that specific members of the Gammaproteobacteria and Alphaproteobacteria were relatively enriched in nodules compared to the water column and surrounding sediments. In particular, members of *Pseudoalteromonas* and *Alteromonas*, which have been hypothesized to play a role in Mn-oxidizing, were found in high relative abundances in microbial assemblages associated with nodules in three different Pacific Ocean gyres (Wu et al., 2013; Blöthe et al., 2015). Relatives of these Gammaproteobacteria, such as *Pseudomonas putida* GB-1, contain two multicopper oxidases and thus have the potential to oxidize both Mn(II) and Mn(III) (Geszvain et al., 2013). In addition, various *Shewanella* strains closely related to OTUs found in the present study can reduce Mn under low O_2_ conditions, where oxidized Mn is used as a terminal electron acceptor, and therefore have the functional capacity for cycling Mn (Wright et al., 2016). Collectively, genetic repertoires catalyzing oxidation-reduction reactions involving Mn-cycling may allow specific gammaproteobacterial OTUs to be successful in the polymetallic-nodule habitat.

Analysis of bacterial distribution patterns revealed that the water column shared 5,861 and 6,827 OTUs with the sediment and nodule habitats, respectively. A much larger number of OTUs (*n* = 29,489), however, were shared between the sediment and nodule habitats. Nevertheless, these results suggest that there is a transfer of certain OTUs from the water column to the benthos.

Our classification of OTUs into “specialists,” “moderate generalists,” and “generalists,” highlighted bacterial populations with specific habitat preferences. Mining operations are predicted to release sediment plumes into the surrounding water (Oebius et al., 2001; Rolinski et al., 2001). Our results suggest that OTUs naturally restricted to the sediments, such as those affiliated with Planctomycetes and Chloroflexi, could be injected into the water column during mining operations during the creation of sediment plumes. Intriguingly, many members of the Planctomycetes and Chloroflexi are restricted to suboxic conditions (Sekiguchi et al., 2003; Elshahed et al., 2007; Fuerst and Sagulenko, 2011), yet OTUs clustering among these organisms were found in the well-oxygenated sediment layers. Both Chloroflexi and Planctomycetes OTUs appeared equally distributed among sediment layers, with a tendency for greater relative abundances of Chloroflexi OTUs in the deepest sediment layer (15–18 cm).

Nodule specialists were widespread among the different sampling strata and APEI-6, and were found at >90% of all sampled sites. Our data show that OTUs affiliated with the Alphaproteobacteria family *Rhodobiaceae* and members of the Gammaproteobacteria *Vibrio* had the widest distributions across these habitats. Similarly, nodule-associated communities were remarkably similar across the Pacific Ocean (Wu et al., 2013). Collectively, this suggests that the composition of bacterial communities is stable and widespread in abyssal polymetallic-nodule habitats.

The most abundant “generalist” OTUs included those affiliated with the cyanobacteria *Prochlorococcus* spp. and *Synechococcus* spp., the SAR324 clade of the Deltaproteobacteria, as well as those most similar to *Candidatus* Actinomarina. Recent studies have revealed that members of the SAR324 clade are metabolically versatile, with the capacity for lithotrophy and heterotrophy (Sheik et al., 2014). Such metabolic adaptations may facilitate the widespread distribution of these organisms, including their ability to inhabit seawater, sediment, and nodule habitats. *Candidatus* Actinomarina is a ubiquitous, ultra-small Actinobacteria with a highly streamlined genome (Ghai et al., 2013). Metagenomic reconstructions of planktonic marine *Actinobacteria* suggest these organisms can rely on photoheterotrophy (including rhodopsin-encoding genes), or obtain energy via oxidation of carbon monoxide, dimethylsulfopropionate, and C2 organic substrates (Mizuno et al., 2015). Overall, OTUs affiliated with the gammaproteobacterial JTB255 and Alphaproteobacteria belonging to the *Rhodospirillaceae* family had the widest distributions, but these OTUs were less abundant or absent in water-column samples. Collectively, our data highlights that the most common specialist OTUs within each habitat distribution group contributed only a small fraction (typically <0.1%) to the total sequence count, regardless of the number of OTUs, except for the nodule specialists that contributed to 5% of total sequences. Hence, the high alpha diversity observed in the nodule and sediment habitats consisted mostly of rare OTUs (<0.001% in relative abundance). In agreement, alpha diversity in similar deep-sea environments (e.g., the North Atlantic; Sogin et al., 2006) and in global ocean transects (Zinger et al., 2011; Salazar et al., 2016) indicates that rare taxa are important contributors to these communities. Our results also indicate that moderate generalists shared between water column and nodule samples were disproportionate contributors to relative abundances compared to the specialist and “generalists” groups.

One of the most intriguing results of our examination of microorganisms associated with seawater, sediments, and polymetallic nodules in the CCZ was that particular bacterial OTUs were found in both seawater and nodule samples, albeit in low relatively abundances, but were not retrieved from the sediments. Such observations suggest that certain groups of microorganisms, including those typically found in epipelagic waters, can be present in the benthic realm and in some cases exhibit a strong association with nodules. To our knowledge, this is the first time that OTUs assigned to picoplanktonic cyanobacteria, including those clustering among *Prochlorococcus* and *Synechococcus*, have been found associated with deep-sea nodules. Cyanobacteria have been reported to be transported to the bathypelagic waters and abyssal ocean floor with sinking particles (phyodetritus and fecal pellets; reviewed by Beaulieu, 2002) in in the Northeast Atlantic (Billett et al., 1983; Lampitt, 1985; Rice et al., 1986; Bett et al., 2001) and at a site near the CCZ (Smith et al., 1994). Phytodetritus deposits on the seafloor in the mid-oceanic northeastern Atlantic contained a rich community of active bacteria and cyanobacteria (Lochte and Turley, 1988; Thiel et al., 1990). Pfannkuche and Lochte (1993) report the presence of cyanobacteria in salp fecal pellets on the abyssal ocean floor and in the guts of deep-sea holothurians. Similarly, *Prochlorococcus* has been found in relatively high abundances in mesopelagic waters, a finding attributed to physical mixing of water and transport of sinking particles (Jiao et al., 2014). *Prochlorococcus* OTUs have also been retrieved from deep-sea hydrothermal vents (Huber et al., 2007), at depths >700 m in the North Pacific Subtropical Gyre (Pham et al., 2008; Brown et al., 2009), and at >2,000 m (Jing et al., 2013; Tseng et al., 2015) in the South China Sea. Richardson and Jackson (2007) utilized inverse and network analyses of food webs in the Equatorial Pacific and Arabian Sea to highlight the disproportionate role of surface picoplankton to deep-sea export. In a study of eukaryotic 18S rRNA gene amplicons from UK-1 Stratum A, Shulse et al. (2016) noted that photosynthetic eukaryotes (e.g., *Cryptophyceae* and *Archaeaplastida*) were relatively enriched on nodules. Together, these studies suggest an important role for these picoplanktonic organisms in the export of material to the benthos (see also Bienhold et al., 2012; Durkin et al., 2015). However, our data shed light on specific populations, including members of the cyanobacteria, that may contribute to downward sinking phytodetritus and become associated with deep-sea nodules.

It is important to consider whether enrichment of certain bacterial taxa (including *Prochlorococcus*) typically found in the upper ocean could result from contamination of nodule samples by water column dwelling organisms. For those samples collected using a boxcore, such contamination may have occurred during the corer's ascent. However, the majority of nodule samples analyzed in the current study (only 7 out of 89 were collected with a boxcorer) were collected using a megacorer, which seals in bottom water and sediments upon collection at the seafloor. Hence, contamination of nodules collected using megacore sampling is much less likely. Moreover, upon collection, nodules were rinsed with 0.2 μm filtered near-bottom water, and prior to DNA extraction, nodules were rinsed again with autoclaved 0.2 μm filtered near-bottom waters. With these efforts to minimize possible contamination, we suspect our results indicating epipelagic bacteria can be selectively associated with nodules are robust.

Several possible mechanisms could explain these observations. In particular, we sought to test the hypothesis that specific bacteria might become associated (e.g., via ingestion) with other nodule-dwelling organisms using network analyses to evaluate the frequency of co-occurring eukaryotic 18S rRNA and bacterial 16S rRNA OTUs. This network analysis revealed highly significant correlations between the occurrences of particular foraminiferal and bacterial OTUs (Figure S6). Diverse sessile foraminiferal assemblages are almost always present on nodule exteriors (Gooday et al., 2015), and sometimes in the interiors of nodules (Maybury, 1996). The vast majority of foraminifera that encrust nodule surfaces in the UK-1 Stratum A (Gooday et al., 2015), and elsewhere in the North Pacific (Mullineaux, 1987; Veillette et al., 2007), are exclusive to this habitat and not present in the sediment. Some shallow-water foraminifera feed selectively on bacteria (Lee et al., 1991; Langezaal et al., 2005; Mojtahid et al., 2011), and it is likely that deep-sea foraminifera do so as well (Gooday et al., 2008). Hence, it is possible that our findings reflect the consumption of specific bacterial taxa by nodule-associated foraminifera. A related possibility is that nodule-associated foraminifera are actively feeding on phytodetritus derived from the euphotic zone. This is a well-established mode of feeding for certain opportunistic calcareous species in the deep sea, including those living at abyssal depths (Gooday, 1988, 1993; Gooday et al., 2008). Since cyanobacteria are a known component of phytodetritus (Thiel et al., 1990), this could explain the association of cyanobacterial OTUs (including those assigned to *Prochlorococcus* and *Synechococcus*) with nodules from UK-1 Stratum A. However, although calcareous foraminifera have been found on nodules, they are generally minor components of these assemblages (Mullineaux, 1987; Veillette et al., 2007; Gooday et al., 2015). In fact, all of the foraminifera OTUs identified in the network analysis (except for the agglutinated *Trochammina* sp.) clustered among shallow-water calcareous species, the majority of them clustering among so-called “larger foraminifera” that host algal symbionts and are generally found in shallow-water reefs. Hence, many of the foraminiferal sequences obtained from the nodules may have derived from dormant propagules (Alve and Goldstein, 2010) transported from the upper ocean on sinking detrital aggregates. However, our genetic analyses were unlikely to recognize the kinds of foraminifera (e.g., komokiaceans) that dominate nodule-encrusting communities since it is difficult to extract DNA and hence retrieve sequence information from these organisms (Lecroq et al., 2009). It therefore remains possible that the association of cyanobacterial OTUs with nodules may reflect foraminiferal consumption of these groups.

Another possible explanation for the highly correlated relationship in nodules but not in sediments between shallow-water foraminiferal OTUs and cyanobacteria from the upper water column may be that nodules selectively retain particles due to their porous structure (see e.g., Blöthe et al., 2015). This mechanism could protect cyanobacterial cells from destruction by the deposit-feeding megafaunal organisms that typically consume seafloor deposits of phytodetritus (Smith et al., 1996; Bett et al., 2001). As such, nodule retention of particles may have a largely unexplored consequence on deep-sea carbon cycling by protecting phytodetritus rich in the energy and nutrients that is actively consumed by many metazoans (Smith et al., 1996). The selective preservation of phytodetritus in nodules could impact how this energy is processed, specifically shifting its use toward meiofaunal and microfaunal organisms.

The results presented in the present paper raise important questions, such as (i) mechanisms of phytodetritus export to polymetallic nodules, (ii) turnover of phytodetrital matter in deep-sea sediments and polymetallic nodule fields, (iii) role of enriched cyanobacteria, such as *Prochlorococcus* in the nodule fields, and (iv) whether the detected OTUs found in both the water column and polymetallic nodules are active in the deep-sea or not. These questions all have implications for our understanding of the impact of planktonic export from the water column to the structure and function of communities dwelling in deep-sea sediments and nodules. Targeted metagenomics and metatranscriptomics may help solve some of these questions, in particular the latter question to determine the activity and influence of photic zone bacterioplankton populations in the deep-sea.

## Conclusions

Our results significantly expand recent advances in understanding microbial ecology and biogeography in abyssal manganese nodule fields, by including a wider area of the CCZ at high spatial resolution. In addition, we explored underlying patterns in the distributions of bacterial taxa in the water column, sediments, and nodules. Our findings highlight key differences in the distributions of specific bacterial assemblages in abyssal habitats. Overall, these results may provide a new set of tools for monitoring ecosystem impacts associated with deep-sea polymetallic-nodule mining, in particular monitoring the injection of specialized sediment bacterial OTUs into the water column for tracking the dispersal and ecological effects of mining plumes.

## Author contributions

CRS and MC conceived the study and designed research; ML, BM, AG, DA, CRS, and MC performed research; ML, BM, and MC analyzed data, and all authors contributed to writing of the manuscript.

### Conflict of interest statement

The authors declare that the research was conducted in the absence of any commercial or financial relationships that could be construed as a potential conflict of interest.
