# Supplementary material for: From the Surface to the Deep-Sea: Bacterial Distributions across Polymetallic Nodule Fields in the Clarion-Clipperton Zone of the Pacific Ocean
Source: Front Microbiol. 2017 Sep 8;8:1696. doi: 10.3389/fmicb.2017.01696 (PMC5596108; doi:10.3389/fmicb.2017.01696)
Supplement: Supplementary file 1 [file DataSheet1.docx]

**Supporting information**

**Supplementary Figures and Tables**

**Figure S1.** Alpha diversity indices in different vertical segments (i.e. water column zones, sediment layers and nodule samples) among all samples (A), and nodule samples collected in AB02 (B).

**Figure S2.** Relative abundances of Chloroflexi (A), and Planctomycetes (B), distributed over the different sediment layers in all samples. The position of individual OTUs has been offset on the x- and y-axis to reduce over-plotting.

**Figure S3.** Least-squares linear regression analyses of relative abundances between OTUs in different vertical segments and in the nodule habitat.

**Figure S4.** Distribution of total number of sequences of OTUs affiliated with cyanobacteria in different vertical segments (A), and distribution of the most abundant cyanobacterial OTUs across all other samples. The depth position in (B) has been offset to reduce over plotting and is thus approximate.

**Figure S5.** Distribution of abundant OTUs that reached >1% in relative abundance in the epipelagic, nodule and sediment layers. Arrows denote distribution of OTUs found in both the epipelagic and nodule and/or sediment layers. Positions of OTUs have been offset to reduce over-plotting and depth positions are approximate.

**Figure S6.** Network diagram of bacterial OTUs and foraminiferal species from 16S rRNA and 18S rRNA sequences obtained during AB01 (Shulse et al., 2016). Each line "edge" in the network represents a highly significant correlation between the relative abundances of foraminiferal 18S rRNA OTUs and 16S rRNA OTUs. Each graph depict a network with significant co-occurrences found between foraminiferal 18S rRNA OTUs and bacterial 16S rRNA OTUs.

**Table S1.** Summary of samples obtained in the AB-02 cruise. A total of 585 sediment, nodule, and water samples for subsequent microbial diversity analyses were collected from 10 CTD rosette casts (4 from UK-1, 5 from OMS and 1 from APEI-6), 25 megacore deployments (13 in UK-1, 12 in OMS), and 28 boxcore deployments (14 in UK-1, 12 in OMS and 2 in APEI-6).

| Type of sampling | Claim | Number of samples | Number of stations sampled | | Water samples | Sediment samples | Nodule samples |
| --- | --- | --- | --- | --- | --- | --- | --- |
| CTD | APEI-6 | 8 | | 2 | 8 | 0 | 0 |
| Megacore | APEI-6 | 0 | | 0 | 0 | 0 | 0 |
| Boxcore | APEI-6 | 6 | | 2 | 0 | 4 | 4 |
| CTD | UK-1B | 32 | | 4 | 32 | 0 | 0 |
| Megacore | UK-1B | 230 | | 12 | 0 | 171 | 59 |
| Boxcore | UK-1B | 30 | | 9 | 0 | 0 | 30 |
| CTD | OMS | 40 | | 5 | 40 | 0 | 0 |
| Megacore | OMS | 216 | | 12 | 0 | 164 | 52 |
| Boxcore | OMS | 21 | | 11 | 0 | 0 | 21 |

**Table S2.** Classification of OTUs into “specialists”, “moderate generalists” and “generalists” according to presence/absence patterns in the three habitats (water column, sediment and nodules) and eight segments (i.e. the epipelagic, mesopelagic, and bathypelagic zones, the 0-5 cm, 5-6 cm, 6-8 cm, 8-10 cm, and 15-18 cm sediment layers and nodules). Data are provided for the top ten most abundant OTUs within each classification and water column zones, sediment layer and nodule samples. The centroid sequence for each OTU was manually blasted (blastn; GenBank) and the best clone and genome hit is provided with accession number and % match. Average relative abundances (% of total sequences) are provided with standard deviation and maximum relative abundance detected. OTUs are sorted according to average relative abundance within each classification of habitats or combination of habitats and/or vertical distributions in the water column zones, sediment layers and nodule samples.

| **Habitat** | **OTU** | **Phyla/Class** | **Taxa** | **Blastn best clone and complete genome hit** | **Accession number and % match** | **Avg. Rel. Abund (% of total sequences) ± SD and maximum** |
| --- | --- | --- | --- | --- | --- | --- |
|  | 11413 | Actinobacteria | *Candidatus*_Actinomarina | Uncultured bacterium clone GAL_5C7 | KC197665.1 (99) | 0.004±0.09 (1.96) |
|  |  |  |  | *Actinobacteria* bacterium IMCC26256 | CP011489.1 (85) |  |
|  | 22420 | Alphaproteobacteria | OCS116_clade | Uncultured marine bacterium clone BIOS04_GYR_180m34 | KM222851.1 (99) | 0.003±0.07 (1.51) |
|  |  |  |  | *Candidatus* Phaeomarinobacter ectocarpi | HG966617.1 (91) |  |
|  | 14263 | Planctomycetes | *Rhodopirellula* | Uncultured bacterium clone BF2010_Nov_21m_B5 | JX864593.1 (99) | 0.002±0.05 (1.21) |
|  |  |  |  | *Pirellula* sp. SH-Sr6A | CP011272.1 (87) |  |
| Epipelagic | 33765 | Cyanobacteria | ML635J-21 | Uncultured bacterium clone N422B_21 | GU940985.1 (99) | 0.002±0.05 (1.09) |
|  |  |  |  | *Alkaliphilus metalliredigens* strain QYMF | NR_074633.1 (86) |  |
| n=81 | 12480 | Cyanobacteria | ML635J-21 | Uncultured bacterium clone N422B_21 | GU940985.1 (98) | 0.002±0.049 (1.03) |
|  |  |  |  | *Alkaliphilus metalliredigens* strain QYMF | NR_074633.1 (86) |  |
| 0.02 % | 13852 | Cyanobacteria | ML635J-21 | Uncultured bacterium clone N422B_21 | GU940985.1 (98) | 0.002±0.05 (1.06) |
|  |  |  |  | *Alkaliphilus metalliredigens* strain QYMF | NR_074633.1 (86) |  |
|  | 12510 | Bacteroidetes | Unclassified | Uncultured marine bacterium clone BIOS04_MAR_15m70 | KM223508.1 (99) | 0.002±0.022 (0.33) |
|  |  |  |  | Uncultured *Sphingobacteria* bacterium | FQ032809.1 (89) |  |
|  | 32171 | Actinobacteria | *Candidatus*_Actinomarina | Uncultured bacterium clone GAL_5C7 | KC197665.1 (99) | 0.002±0.043 (0.91) |
|  |  |  |  | *Actinobacteria* bacterium IMCC26256 | CP011489.1 (85) |  |
|  | 19523 | Planctomycetes | *Rhodopirellula* | Uncultured bacterium clone BF2010_Nov_21m_B5 | JX864593.1 (99) | 0.001±0.041 (0.88) |
|  |  |  |  | *Pirellula* sp. SH-Sr6A | CP011272.1 (87) |  |
|  | 17536 | Actinobacteria | Sva0996_marine_group | Uncultured bacterium clone O4 | GQ377779.1 (99) | 0.001±0.037 (0.78) |
|  |  |  |  | *Actinobacteria* bacterium IMCC26256 | CP011489.1 (89) |  |
|  | 7734 | Deltaproteobacteria | *Nitrospina* | Uncultured delta proteobacterium clone JL-ETNP-R9 | AY726827.1 (99) | 0.004±0.037 (0.46) |
|  |  |  |  | *Mahella australiensis* 50-1 BON | CP002360.1 (85) |  |
|  | 12534 | Deltaproteobacteria | *Nitrospina* | Uncultured delta proteobacterium clone JL-ETNP-R9 | AY726827.1 (99) | 0.003±0.029 (0.37) |
|  |  |  |  | *Clostridium acidurici* 9a | CP003326.1 (86) |  |
|  | 10010 | Acidobacteria | Subgroup_6 | Uncultured bacterium clone JdFBHP37 | JQ678351.1 (99) | 0.003±0.024 (0.28) |
|  |  |  |  | *Acidobacteria* bacterium DSM 100886 | CP015136.1 (99) |  |
| Mesopelagic | 9470 | Deltaproteobacteria | Unclassified | Uncultured bacterium clone: OTU060_Ref_Clone02 | AB694330.1 (98) | 0.002±0.019 (0.24) |
|  |  |  |  | *Alkaliphilus metalliredigens* QYMF | CP000724.1 (85) |  |
| n=215 | 26012 | Firmicutes | 64K2 | Uncultured *Poribacteria* bacterium clone HF770_17N21 | EU361159.1 (99) | 0.002±0.017 (0.17) |
|  |  |  |  | *Paenibacillus* sp. FSL R7-0331 | CP009284.1 (81) |  |
| 0.06% | 26797 | Deltaproteobacteria | Sh765B-TzT-29 | Uncultured bacterium clone F9P262000_S_I20 | HQ674477.1 (98) | 0.002±0.012 (0.16) |
|  |  |  |  | *Desulfohalobium retbaense* DSM 5692 | CP001734.1 (87) |  |
|  | 17200 | Planctomycetes | OM190 | Uncultured bacterium clone JdFBHP56 | JQ678369.1 (99) | 0.002±0.013 (0.16) |
|  |  |  |  | *Kuenenia stuttgartiensis* genome fragment KUST_E | CT573071.1 (81) |  |
|  | 19369 | Gammaproteobacteria | *Alcanivorax* | *Alcanivorax venustensis* strain 2PR57-5 | EU440993.1 (99) | 0.002±0.014 (0.68) |
|  |  |  |  | *Alcanivorax dieselolei* B5 | CP003466.1 (98) |  |
|  | 26135 | Marinimicrobia | SAR406 clade | Uncultured bacterium clone 41-12-21 | JN018770.1 (99) | 0.002±0.014 (0.12) |
|  |  |  |  | *Syntrophus aciditrophicus* SB | CP000252.1 (83) |  |
|  | 12009 | Actinobacteria | *Acidimicrobiales* | Uncultured bacterium clone JL-ETNP-S56 | AY726867.1 (99) | 0.002±0.014 (0.17) |
|  |  |  |  | *Actinobacteria* bacterium IMCC26256 | CP011489.1 (92) |  |
|  | 4165 | Lentisphaerae | R76-B128 | Uncultured bacterium clone FS266-26B-03 | DQ513076.1 (99) | 0.011±0.091 (1.04) |
|  |  |  |  | *Verrucomicrobia* bacterium L21-Fru-AB | CP010904.1 (86) |  |
|  | 9525 | Lentisphaerae | R76-B128 | Uncultured bacterium clone FS266-26B-03 | DQ513076.1 (99) | 0.006±0.050 (0.51) |
|  |  |  |  | *Verrucomicrobia* bacterium L21-Fru-AB | CP010904.1 (86) |  |
|  | 7187 | Marinimicrobia | SAR406_clade | Uncultured bacterium clone FS142-10B-02 | DQ513043.1 (99) | 0.006±0.037 (0.37) |
| Bathypelagic |  |  |  | *Candidatus* Babela massiliensis strain BABL1 | HG793133.1 (82) |  |
|  | 4805 | Lentisphaerae | R76-B128 | Uncultured bacterium clone FS266-26B-03 | DQ513076.1 (99) | 0.006±0.049 (0.47) |
| n=500 |  |  |  | *Verrucomicrobia* bacterium L21-Fru-AB | CP010904.1 (86) |  |
|  | 6077 | Lentisphaerae | R76-B128 | Uncultured bacterium clone FS266-26B-03 | DQ513076.1 (99) | 0.005±0.040 (0.52) |
| 0.27% |  |  |  | *Verrucomicrobia* bacterium L21-Fru-AB | CP010904.1 (86) |  |
|  | 8271 | Marinimicrobia | SAR406 clade | Uncultured bacterium clone FS142-10B-02 | DQ513043.1 (99) | 0.004±0.027 (0.28) |
|  |  |  |  | *Candidatus* Babela massiliensis strain BABL1 | HG793133.1 (82) |  |
|  | 11265 | Deltaproteobacteria | SAR324 clade (Marine_group_B) | Uncultured SAR324 cluster bacterium clone PRTBB8646 | HM799049.1 (99) | 0.004±0.023 (0.17) |
|  |  |  |  | *Rickettsiales* bacterium Ac37b | CP009217.2 (85) |  |
|  | 22775 | Marinimicrobia | SAR406_clade | Uncultured bacterium clone FS142-10B-02 | DQ513043.1 (99) | 0.004±0.025 (0.24) |
|  |  |  |  | *Candidatus* Babela massiliensis strain BABL1 | HG793133.1 (82) |  |
|  | 7714 | Gammaproteobacteria | E01-9C-26 marine group | Uncultured deep-sea bacterium clone Lau62_Abe | KP005052.1 (99) | 0.004±0.022 (0.22) |
|  |  |  |  | *Thiolapillus brandeum* | AP012273.1 (91) |  |
|  | 7819 | Acidobacteria | Subgroup_6 | Uncultured *Acidobacteria* bacterium HF4000_26D02 | GU474896.1 (99) | 0.004±0.030 (0.49) |
|  |  |  |  | *Acidobacteria* bacterium DSM 100886 | CP015136.1 (90) |  |

**Table S2 cont.** Sediment specialists.

| **Habitat** | **OTU** | **Phyla/Class** | **Taxa** | **Blastn best clone and complete genome hit** | **Accession number and % match** | **Avg. Rel. Abund (% of total sequences) ± SD and maximum** |
| --- | --- | --- | --- | --- | --- | --- |
|  | 024538 | Parcubacteria | Unclassified | Uncultured planctomycete clone JK249 | DQ368332.2 (94) | 0.001±0.031 (0.66) |
|  |  |  |  | *Parcubacteria (Campbellbacteria)* bacterium GW2011_OD1_34_28 | CP011215.1 (87) |  |
|  | 037254 | Deltaproteobacteria | *Nitrospinaceae* | Uncultured bacterium clone NCB192 | JX227229.1 (99) | 0.0009±0.009 (0.18) |
|  |  |  |  | *Pelobacter carbinolicus* DSM 2380 | CP000142.2 (88) |  |
|  | 042275 | Deltaproteobacteria | GR-WP33-30 | Uncultured bacterium clone SPG12_213_223_B51 | FJ746219.1 (98) | 0.0007±0.004 (0.06131) |
| Sediment |  |  |  | *Anaeromyxobacter dehalogenans* 2CP-C | CP000251.1 (87) |  |
| 0-5 cm | 024598 | Chloroflexi | SAR202 clade | Uncultured bacterium clone PC-C158 | FJ938708.1 (95) | 0.0007±0.0040 (0.04133) |
|  |  |  |  | *Dehalogenimonas* sp. WBC-2 | CP011392.1 (87) |  |
| n=141 | 023161 | Alphaproteobacteria | S26-47 | Uncultured alpha proteobacterium clone: MCD_AB11_123cm_B42 | LC050100.1 (99) | 0.0006±0.0043 (0.05435) |
|  |  |  |  | *Devosia* sp. H5989 | CP011300.1 (92) |  |
| 0.015% | 023344 | Planctomycetes | CCM11a | Uncultured deep-sea bacterium clone Ucm1561 | AM997286.1 (99) | 0.0005±0.0085 (0.1782) |
|  |  |  |  | *Phycisphaera mikurensis* NBRC 102666 | AP012338.1 (80) |  |
|  | 038475 | Acidobacteria | *Acidobacteria* | Uncultured delta proteobacterium clone ES0303-B69 | FJ437697.1 (92) | 0.0005± 0.0032 (0.03401) |
|  |  |  |  | *Acidobacterium capsulatum* ATCC 51196 | CP001472.1 (84) |  |
|  | 036110 | Chloroflexi | SAR202 clade | Uncultured deep-sea bacterium clone Ulrdd_29 | AM997493.1 (98) | 0.0004±0.0040 (0.06775) |
|  |  |  |  | *Dictyoglomus turgidum* DSM 6724 | CP001251.1 (85) |  |
|  | 042070 | Parcubacteria | Unclassified | Uncultured candidate division OP11 bacterium MERTZ_2CM_148 | AF424438.1 (96) | 0.0004±0.0076 (0.15655) |
|  |  |  |  | *Parcubacteria (Wolfebacteria)* bacterium GW2011_GWB1_47_1 | CP011209.1 (86) |  |
|  | 021567 | Chloroflexi | S085 | Uncultured bacterium clone EP2-23 | EF491481.1 (99) | 0.0004±0.0030 (0.03785) |
|  |  |  |  | Uncultured bacterium 5G4 | KT342856.1 (92) |  |
|  | 040614 | Candidate_division_OP3 Unclassified | | Uncultured bacterium clone BR54_2_033_4650_3_13to16_spongegarden_158.1 | KT223301.1 (91) | 0.0005±0.004 (0.07) |
|  |  |  |  | *Thermodesulfatator indicus* DSM 15286 | CP002683.1 (81) |  |
|  | 040358 | Hydrogenedentes | | Uncultured bacterium clone: MCD_AB11_65cm_B42 | LC050074.1 (99) | 0.0004±0.002 (0.02) |
|  |  |  |  | *Geoalkalibacter subterraneus* strain Red1 | CP010311.1 (86) |  |
|  | 044069 | Unclassified | Unclassified | Uncultured bacterium clone st96 | FR734402.1 (88) | 0.0003±0.004 (0.07) |
| Sediment |  |  |  | Uncultured bacterium 293 | GU260698.1 (83) |  |
| 5-6 cm | 042656 | Alphaproteobacteria | *Rhodospirillaceae* | Uncultured bacterium clone SBB21 | JX227416.1 (98) | 0.0003±0.002 (0.03) |
|  |  |  |  | Uncultured alpha proteobacterium HF0130_06E21 | GU474868.1 (92) |  |
| n=82 | 042967 | Alphaproteobacteria | *Rhodospirillaceae* | Uncultured bacterium clone SBB21 | JX227416.1 (99) | 0.0002±0.0022 (0.02) |
|  |  |  |  | Uncultured alpha proteobacterium HF0130_06E21 | GU474868.1 (93) |  |
| 0.005% | 035326 | Chloroflexi | KD4-96 | Uncultured bacterium clone pTVGB25 | GU196044.1 (98) | 0.0002±0.002 (0.02) |
|  |  |  |  | Uncultured bacterium clone WBA-13 | JQ180422.1 (86) |  |
|  | 046294 |  |  | Uncultured bacterium clone GBc137 | JQ612289.1 (99) | 0.0002±0.0023 (0.02) |
|  |  |  |  | Uncultured *Acidobacteria* bacterium clone 41b15 | AY281356.1 (97) |  |
|  | 043237 | Planctomycetes | Pla4_lineage | Uncultured *planctomycete* clone MS-A121 | FJ949400.1 (95) | 0.0002±0.0026 (0.04) |
|  |  |  |  | *Kuenenia stuttgartiensis* genome fragment KUST_E | CT573071.1 (82) |  |
|  | 040936 | Planctomycetes | OM190 | Uncultured bacterium clone AC3-7 | KJ590543.1 (94) | 0.0002±0.0018 (0.02) |
|  |  |  |  | *Desulfovibrio alaskensis* G20 | CP000112.1 (84) |  |
|  | 039075 | Planctomycetes | *Planctomyces* | Uncultured bacterium clone: smkt_Pla_161_001 | AB807253.1 (98) | 0.0001±0.0033 (0.06) |
|  |  |  |  | Uncultured *Planctomycetales* bacterium HF0500_40D21 | GU474923.1 (88) |  |
|  | 012771 | Chloroflexi | S085 | Uncultured bacterium clone EP2-23 | EF491481.1 (99) | 0.001±0.0068 (0.07) |
|  |  |  |  | *Dehalogenimonas* sp. WBC-2 | CP011392.1 (86) |  |
|  | 031244 | Acidobacteria | *Acidobacteria* | Uncultured delta proteobacterium clone ES0303-B69 | FJ437697.1 (92) | 0.001±0.0072 (0.10) |
|  |  |  |  | *Acidobacterium capsulatum* ATCC 51196 | CP001472.1 (85) |  |
|  | 020671 | Planctomycetes | OM190 | Uncultured deep-sea bacterium | AM997545.1 (98) | 0.001±0.0081 (0.11) |
| Sediment |  |  |  | *Kuenenia stuttgartiensis* genome fragment KUST_E | CT573071.1 (82) |  |
| 8-10 cm | 029052 | Chloroflexi | SAR202 clade | Uncultured bacterium clone: 52B1-030 | LC081114.1 (99) | 0.001±0.0063 (0.07) |
|  |  |  |  | *Caldilinea aerophila* DSM 14535 = NBRC 104270 | AP012337.1 (85) |  |
| n=337 | 027139 | Deltaproteobacteria | GR-WP33-30 | Uncultured delta proteobacterium clone B4G | FJ205197.1 (99) | 0.0009±0.0090 (0.17) |
|  |  |  |  | *Desulfuromonas* sp. DDH964 | CP015080.1 (87) |  |
| 0.07% | 024940 | Gemmatimonadetes | *Gemmatimonadaceae* | Uncultured bacterium clone RS-B63 | JF809765.1 (98) | 0.0009±0.0070 (0.12) |
|  |  |  |  | *Geoalkalibacter subterraneus* strain Red1 | CP010311.1 (83) |  |
|  | 017152 | Deltaproteobacteria | GR-WP33-30 | Uncultured delta proteobacterium clone B4G | FJ205197.1 (99) | 0.0009±0.0077 (0.13) |
|  |  |  |  | *Desulfuromonas* sp. DDH964 | CP015080.1 (87) |  |
|  | 015612 | Alphaproteobacteria | *Rhodospirillaceae* | Uncultured alpha proteobacterium clone: MCD_AB11_12.5cm_B35 | LC050035.1 (99) | 0.0009±0.0049 (0.04) |
|  |  |  |  | *Methyloceanibacter caenitepidi* strain: Gela4 | AP014648.1 (91) |  |
|  | 020641 | Acidobacteria | Subgroup_2 | Uncultured *Acidobacteria* bacterium clone 16G | JN178895.1 (96) | 0.0009±0.0047 (0.04) |
|  |  |  |  | *Acidobacterium capsulatum* ATCC 51196 | CP001472.1 (82) |  |
|  | 038952 | Planctomycetes | *Candidatus*_Scalindua | Uncultured bacterium clone SPG12_213_223_B33 | FJ746210.1 (99) | 0.0008±0.0095 (0.16) |
|  |  |  |  | Uncultured bacterium OMZ60_11L23 | EU795287.1 (94) |  |

**Table S2 cont.** Nodule specialists.

| **Habitat** | **OTU** | **Phyla/Class** | **Taxa** | **Blastn best clone and complete genome hit** | **Accession number and % match** | **Avg. Rel. Abund (% of total sequences) ± SD and maximum** |
| --- | --- | --- | --- | --- | --- | --- |
|  | 223 | Nitrospirae | *Nitrospira* | Uncultured bacterium clone CE-6 | HM101002.1 (99) | 0.022±0.105 (1.31) |
|  |  |  |  | *Nitrospira* sp. ENR4 genome assembly NiCh1 | LN885086.1 (89) |  |
|  | 220 | Alphaproteobacteria | *Rhodobiaceae* | Uncultured bacterium clone P0X4b3B06 | EU491404.1 (99) | 0.021±0.076 (0.78) |
|  |  |  |  | *Methyloceanibacter caenitepidi* strain: Gela4 | AP014648.1 (94) |  |
|  | 212 | Deltaproteobacteria | SAR324_clade(Marine_group_B) | Uncultured bacterium clone NAB7 | JX226721.1 (99) | 0.021±0.069 (0.68) |
|  |  |  |  | Uncultured delta proteobacterium HF0070_07E19 | GU474908.1 (88) |  |
| Nodule | 204 | Actinobacteria | OM1_clade | Uncultured bacterium clone UB-68 | KM454253.1 (99) | 0.018±0.093 (1.01) |
|  |  |  |  | *Micromonospora echinaurantiaca* strain DSM 43904 genome assembly | LT607750.1 (88) |  |
| n=4934 | 262 | Proteobacteria | Unclassified | Uncultured bacterium clone P0X4b2A01 | EU491461.1 (99) | 0.013±0.057 (0.71) |
|  |  |  |  | *Woeseia oceani* strain XK5 | CP016268.1 (96) |  |
| 5% | 477 | Deltaproteobacteria | SAR324_clade(Marine_group_B) | Uncultured bacterium clone NAB7 | JX226721.1 (98) | 0.012±0.053 (0.47) |
|  |  |  |  | Uncultured delta proteobacterium HF0070_07E19 | GU474908.1 (89) |  |
|  | 520 | Nitrospirae | *Nitrospira* | Uncultured bacterium clone CE-6 | HM101002.1 (99) | 0.011±0.062 (0.81) |
|  |  |  |  | *Nitrospira* sp. ENR4 genome assembly NiCh1 | LN885086.1 (89) |  |
|  | 644 | Deltaproteobacteria | SAR324_clade(Marine_group_B) | Uncultured bacterium clone NAB7 | JX226721.1 (98) | 0.011±0.037 (0.40) |
|  |  |  |  | Uncultured delta proteobacterium HF0070_07E19 | GU474908.1 (89) |  |
|  | 283 | Alphaproteobacteria | *Rhodobiaceae* | Uncultured bacterium clone P0X4b3B06 | EU491404.1 (99) | 0.010±0.039 (0.39) |
|  |  |  |  | *Methyloceanibacter caenitepidi* strain: Gela4 | AP014648.1 (94) |  |
|  | 745 | Nitrospirae | *Nitrospira* | Uncultured bacterium clone CE-6 | HM101002.1 (99) | 0.010±0.055 (0.68) |
|  |  |  |  | *Nitrospira* sp. ENR4 genome assembly NiCh1 | LN885086.1 (89) |  |

**Table S2 cont. “**Generalists” and “moderate generalists”.

| **Habitat** | **OTU** | **Phyla/Class** | **Taxa** | **Blastn best clone and complete genome hit** | **Accession number and % match** | **Avg. Rel. Abund (% of total sequences) ± SD and maximum** |
| --- | --- | --- | --- | --- | --- | --- |
|  | 77 | Cyanobacteria | FamilyI | Uncultured bacterium clone JS4_D01 | KT318698.1 (99) | 0.12±0.573 (4.60) |
|  |  |  |  | *Prochlorococcus* sp. MIT 0604 | CP007753.1 (99) |  |
|  | 318 | Deltaproteobacteria | SAR324_clade (Marine_group_B) | Uncultured deep-sea bacterium clone Lau20_TuiMalila | KP005024.1 (99) | 0.08±0.255 (1.4) |
|  |  |  |  | *Rickettsiales* bacterium Ac37b | CP009217.2 (84) |  |
|  | 301 | Unclassified | Unclassified | Uncultured marine microorganism clone 41224 | FJ598507.1 (99) | 0.06±0.324 (2.48) |
|  |  |  |  | *Prochlorococcus* *marinus* str. MIT 9312 | CP000111.1 (99) |  |
|  | 267 | Cyanobacteria | *Synechococcus* | Uncultured *Synechococcus* sp. clone JL-ETNP-Z50 | AY726960.1 (99) | 0.06±0.280 (2.65) |
| Generalists |  |  |  | *Synechococcus* WH7803 | CT971583.1 (99) |  |
|  | 176 | Cyanobacteria | *Synechococcus* | Uncultured *Synechococcus* sp. clone JL-ETNP-Z50 | AY726960.1 (99) | 0.04±0.188 (1.46) |
| n=54 |  |  |  | *Synechococcus* WH7803 | CT971583.1 (99) |  |
|  | 266 | Cyanobacteria | FamilyI | Uncultured marine microorganism clone 30615 | FJ598505.1 (99) | 0.04±0.208 (1.57) |
| 1% |  |  |  | *Prochlorococcus* sp. MIT 0604 | CP007753.1 (99) |  |
|  | 2134 | Unclassified | Unclassified | Uncultured marine microorganism clone 41224 | FJ598507.1 (99) | 0.038±0.20 (1.81) |
|  |  |  |  | *Prochlorococcus* marinus str. MIT 9312 | CP000111.1 (99) |  |
|  | 308 | Cyanobacteria | *Synechococcus* | Uncultured *Synechococcus* sp. clone JL-ETNP-Z50 | AY726960.1 (99) | 0.033±0.142 (1.14) |
|  |  |  |  | *Synechococcus* WH7803 | CT971583.1 (99) |  |
|  | 1085 | Actinobacteria | *Candidatus*_Actinomarina | Uncultured bacterium clone DS36_B01 | KT318695.1 (99) | 0.031±0.19 (2.59) |
|  |  |  |  | Actinobacteria bacterium IMCC26256 | CP011489.1 (85) |  |
|  | 1725 | Actinobacteria | *Candidatus*_Actinomarina | Uncultured actinobacterium clone CB01B11 | EF471728.1 (99) | 0.029±0.16 (1.65 |
|  |  |  |  | Actinobacteria bacterium IMCC26256 | CP011489.1 (85) |  |
|  | 2086 | Alphaproteobacteria | *Robiginitomaculum* | Uncultured bacterium clone NCB7 | JX227044.1 (99) | 0.002±0.013 (0.15) |
|  |  |  |  | *Hirschia baltica* ATCC 49814 | CP001678.1 (91) |  |
|  | 14130 | Deltaproteobacteria | *Nitrospinaceae* | Uncultured *Nitrospinaceae* bacterium clone T13J-B101 | JN860396.1 (98) | 0.002±0.025 (0.37) |
|  |  |  |  | *Pelobacter carbinolicus* strain DSM 2380 | NR_075013.1 (85) | |
| Epipelagic | 21044 | Deltaproteobacteria | Unclassified | Uncultured *Nitrospinaceae* bacterium clone T13J-B101 | JN860396.1 (98) | 0.001±0.020 (0.30) |
| plus |  |  |  | *Pelobacter carbinolicus* strain DSM 2380 | NR_075013.1 (85) | |
| Nodule | 15743 | Cyanobacteria | FamilyI | *Synechococcus* sp. RCC1026 | JF306723.1 (99) | 0.001±0.008 (0.08) |
|  |  |  |  | *Prochlorococcus* *marinus* str. MIT 9303 | CP000554.1 (99) |  |
| n=163 | 21591 | Deltaproteobacteria | Unclassified | Uncultured *Nitrospinaceae* bacterium clone T13J-B101 | JN860396.1 (97) | 0.001±0.017 (0.28) |
|  |  |  |  | *Pelobacter carbinolicus* strain DSM 2380 | NR_075013.1 (84) | |
| 0.07% | 29952 | Alphaproteobacteria | SAR116_clade | Uncultured bacterium clone SeaWat_30141 | JQ196698.1 (99) | 0.001±0.01 (0.19) |
|  |  |  |  | *Candidatus* Puniceispirillum marinum IMCC1322 | CP001751.1 (90) |  |
|  | 29171 | Gammaproteobacteria | SAR86_clade | Uncultured bacterium clone P2-31 | KP639001.1 (99) | 0.001±0.011 (0.19) |
|  |  |  |  | *Azotobacter chroococcum* NCIMB 8003 | CP010415.1 (90) |  |
|  | 2666 | Cyanobacteria | FamilyI | Uncultured marine microorganism clone 50604 | FJ598533.1 (99) | 0.001±0.0077 (0.09) |
|  |  |  |  | *Prochlorococcus marinus* str. MIT 9211 | CP000878.1 (99) |  |
|  | 45341 | Planctomycetes | *Planctomycetaceae* | Uncultured bacterium clone T9-1_2 | KX097258.1 (99) | 0.001±0.017 (0.36) |
|  |  |  |  | *Rhodopirellula baltica* SH 1 | BX294149.1 (89) |  |
|  | 20710 | Gammaproteobacteria | JTB255_marine_benthic_group | Uncultured prokaryote clone SMB_AA7 | JX569114.1 (99) | 0.001±0.011 (0.1) |
|  |  |  |  | *Woeseia oceani* strain XK5 | CP016268.1 (99) |  |
|  | 213 | Cyanobacteria | FamilyI | Uncultured *Prochlorococcus* sp. clone JL-ETNP-S18 | AY726833.1 (99) | 0.05±0.272 (1.99) |
|  |  |  |  | *Prochlorococcus marinus* str. MIT 9303 | CP000554.1 (99) |  |
|  | 1032 | Deltaproteobacteria | SAR324_clade(Marine_group_B) | Uncultured deep-sea bacterium clone Lau20_TuiMalila | KP005024.1 (99) | 0.044±0.14 (0.91) |
|  |  |  |  | *Rickettsiales* bacterium Ac37b | CP009217.2 (84) |  |
|  | 886 | Unclassified | Unclassified | Uncultured deep-sea bacterium clone Lau230_TahiMoana | KP005043.1 (99) | 0.041±0.152 (1.04) |
|  |  |  |  | *Candidatus* Thioglobus autotrophicus strain EF1 | CP010552.1 (99) |  |
| All Water Column | 680 | Gammaproteobacteria | *Oceanospirillales* | Uncultured bacterium clone A723009 | AY907822.1 (99) | 0.033±0.207 (1.77) |
| plus |  |  |  | *Candidatus* Thioglobus singularis PS1 | CP006911.1 (98) |  |
| Nodule | 694 | Cyanobacteria | FamilyI | Uncultured *Prochlorococcus* sp. clone JL-ETNP-S18 | AY726833.1 (99) | 0.033±0.161 (1.37) |
| n=801 |  |  |  | *Prochlorococcus marinus* str. MIT 9303 | CP000554.1 (99) |  |
|  | 767 | Marinimicrobia_(SAR406_clade) | | Uncultured SAR406 cluster bacterium clone ESP200-K10-15 | DQ810538.1 (99) | 0.032±0.210 (2.56) |
| 2.5% |  |  |  | *Pelobacter propionicus DSM 2379* | CP000482.1 (82) |  |
|  | 2170 | Deltaproteobacteria | SAR324_clade(Marine_group_B) | Delta proteobacterium SCGC AAA240-J17 | HQ675606.1 (99) | 0.029±0.114 (0.86) |
|  |  |  |  | *Rickettsiales bacterium Ac37b* | CP009217.2 (84) |  |
|  | 985 | Gammaproteobacteria | *Oceanospirillales* | Uncultured bacterium clone A723009 | AY907822.1 (99) | 0.025±0.166 (1.69) |
|  |  |  |  | *Candidatus* Thioglobus singularis PS1 | CP006911.1 (98) |  |
|  | 1750 | Deltaproteobacteria | SAR324_clade(Marine_group_B) | Uncultured bacterium clone YD1000-105 | JX441590.1 (99) | 0.025±0.101 (0.76) |
|  |  |  |  | *Rickettsiales bacterium Ac37b* | CP009217.2 (85) |  |
|  | 438 | Cyanobacteria | FamilyI | Uncultured Prochlorococcus sp. clone JL-ETNP-S18 | AY726833.1 (99) | 0.024±0.112 (0.87) |
|  |  |  |  | *Prochlorococcus marinus* str. MIT 9303 | CP000554.1 (99) |  |

**Table S3.** The most prominent OTUs among nodule assemblages with the highest occupancy (number of sites occupied) and best match of centroid 16S rRNA gene sequence in GenBank using blastn with information on source of matched clone and reference. This table summarizes 89 sites from which nodule samples were retrieved.

| **OTU** | **Phyla/Class** | **Taxa** | **Occupancy**  **% of sites** | **blastn** | **Accession number & % match** | **Source of clone** | **Reference** |
| --- | --- | --- | --- | --- | --- | --- | --- |
| 185 | Proteobacteria | Unclassified | 99 | Uncultured *Vibrio* sp. clone E20 | KT336032.1 (99) | Mn nodule | Blöthe,.M., et al. 2015 |
| 194 | Alphaproteobacteria | *Hyphomicrobiaceae* | 97 | Uncultured bacterium clone NBB226 | JX227030.1 (99) | Mn nodule | Wu,Y.-H., et al.2016 |
| 33 | Proteobacteria | Unclassified | 100 | Uncultured *Vibrio* sp. clone E20 | KT336032.1 (99) | Mn nodule | Blöthe,M., et al. 2015 |
| 50 | Gammaproteobacteria | JTB255 marine benthic group | 100 | Uncultured bacterium clone F06 | KT336050.1 (99) | Mn nodule | Blöthe,M., et al. 2015 |
| 196 | Planctomycetes | *Planctomycetaceae* | 97 | Uncultured bacterium clone NBB126 | JX226930.1 (99) | Mn nodule | Wu,Y.-H., et al.2016 |
| 208 | Gammaproteobacteria | JTB255 marine benthic group | 100 | Uncultured bacterium clone F06 | KT336050.1 (99) | Mn nodule | Blöthe,M., et al. 2015 |
| 98 | Alphaproteobacteria | Unclassified | 97 | Uncultured bacterium clone NAB43 | JX226757.1 (99) | Mn nodule | Wu,Y.-H., et al.2016 |
| 1278 | Alphaproteobacteria | *Rhodobiaceae* | 97 | Uncultured bacterium clone SAB94 | JX227371.1 (99) | Mn nodule | Wu,Y.-H., et al.2016 |
| 131 | Proteobacteria | Unclassified | 98 | Uncultured *Vibrio* sp. clone E20 | KT336032.1 (99) | Mn nodule | Blöthe,M., et al. 2015 |
| 222 | Proteobacteria | Unclassified | 98 | Uncultured *Vibrio* sp. clone E20 | KT336032.1 (99) | Mn nodule | Blöthe,M., et al. 2015 |
| 258 | Proteobacteria | Unclassified | 97 | Uncultured *Vibrio* sp. clone E20 | KT336032.1 (99) | Mn nodule | Blöthe,M., et al. 2015 |
| 355 | Alphaproteobacteria | *Rhodobiaceae* | 98 | Uncultured bacterium clone SAB94 | JX227371.1 (99) | Mn nodule | Wu,Y.-H., et al.2016 |
| 121 | Alphaproteobacteria | *Rhodobiaceae* | 98 | Uncultured bacterium clone SAB94 | JX227371.1 (99) | Mn nodule | Wu,Y.-H., et al.2016 |
| 950 | Alphaproteobacteria | Unclassified | 96 | Uncultured bacterium clone NAB43 | JX226757.1 (99) | Mn nodule | Wu,Y.-H., et al.2016 |
| 145 | Gammaproteobacteria | JTB255 marine benthic group | 100 | Uncultured bacterium clone F06 | KT336050.1 (99) | Mn nodule | Blöthe,M., et al. 2015 |
| 2609 | Gammaproteobacteria | JTB255 marine benthic group | 91 | Uncultured bacterium clone SAB86 | JX227363.1 (99) | Mn nodule | Wu,Y.-H., et al.2016 |
| 136 | Betaproteobacteria | *Nitrosomonas* | 100 | Uncultured *Nitrosospira* sp. clone T13J-B93 | JN860390.1 (99) | Low temp. hydrother. | Li,J., et al. 2013 |
| 764 | Gammaproteobacteria | JTB255 marine benthic group | 97 | Uncultured bacterium clone SAB8 | JX227285.1 (99) | Mn nodule | Wu,Y.-H., et al.2016 |
| 604 | Alphaproteobacteria | *Rhodobiaceae* | 97 | Uncultured bacterium clone SAB94 | JX227371.1 (99) | Mn nodule | Wu,Y.-H., et al.2016 |
| 719 | Proteobacteria | Unclassified | 97 | Uncultured *Vibrio* sp. clone E20 | KT336032.1 (99) | Mn nodule | Blöthe,M., et al. 2015 |

**Table S4.** The most prominent OTUs among all communities in all habitats. Occupancy (number of sites occupied) was determined for all claims including APEI-6 and among all habitats. We include best match of centroid 16S rRNA gene sequence in GenBank using blastn with information on source of matched clone and reference Note that ~20 %, ~60% and ~20 % of the samples were obtained from the water column, sediment and nodule habitat, respectively.

| **OTU** | **Phyla/Class** | **Taxa** | **Occupancy**  **% of sites** | **blastn** | **Accession number & % match** | **Source of clone** | **Reference** |
| --- | --- | --- | --- | --- | --- | --- | --- |
| 2 | Gammaproteobacteria | JTB255 marine benthic group | 90 | Uncultured bacterium clone F06 | KT336050.1 (99) | Mn nodule | Blöthe,M., et al. 2015 |
| 4 | Alphaproteobacteria | *Rhodospirillaceae*_uncultured | 90 | Uncultured bacterium clone SCB39 | JX227511.1 (99) | Sediment | Wu,Y.-H., et al.2016 |
| 18 | Gammaproteobacteria | JTB255 marine benthic group | 89 | Uncultured bacterium clone F06 | KT336050.1 (99) | Mn nodule | Blöthe,M., et al. 2015 |
| 16 | Acidobacteria | Subgroup_21 | 89 | Uncultured bacterium clone SCB253 | JX227725.1 (99) | Sediment | Wu,Y.-H., et al.2016 |
| 27 | Alphaproteobacteria | *Rhodospirillaceae*_uncultured | 89 | Uncultured bacterium clone SCB39 | JX227511.1 (99) | Sediment | Wu,Y.-H., et al.2016 |
| 29 | Gammaproteobacteria | JTB255 marine benthic group | 89 | Uncultured bacterium clone F06 | KT336050.1 (99) | Mn nodule | Blöthe,M., et al. 2015 |
| 34 | Alphaproteobacteria | *Rhodospirillaceae*_uncultured | 89 | Uncultured bacterium clone SCB39 | JX227511.1 (99) | Sediment | Wu,Y.-H., et al.2016 |
| 66 | Acidobacteria | Subgroup_21 | 89 | Uncultured bacterium clone SCB253 | JX227725.1 (99) | Sediment | Wu,Y.-H., et al.2016 |
| 24 | Alphaproteobacteria | *Rhodospirillaceae*_uncultured | 88 | Uncultured bacterium clone SCB39 | JX227511.1 (99) | Sediment | Wu,Y.-H., et al.2016 |
| 106 | Gammaproteobacteria | JTB255 marine benthic group | 88 | Uncultured bacterium clone F06 | KT336050.1 (99) | Mn nodule | Blöthe,M., et al. 2015 |
| 94 | Acidobacteria | Subgroup_21 | 88 | Uncultured bacterium clone SCB253 | JX227725.1 (99) | Sediment | Wu,Y.-H., et al.2016 |
| 92 | Acidobacteria | Subgroup_21 | 87 | Uncultured bacterium clone SCB253 | JX227725.1 (99) | Sediment | Wu,Y.-H., et al.2016 |
| 170 | Alphaproteobacteria | *Rhodospirillaceae*_uncultured | 87 | Uncultured bacterium clone SCB39 | JX227511.1 (99) | Sediment | Wu,Y.-H., et al.2016 |
| 31 | Gammaproteobacteria | JTB255 marine benthic group | 86 | Uncultured bacterium clone F06 | KT336050.1 (99) | Mn nodule | Blöthe,M., et al. 2015 |
| 46 | Gammaproteobacteria | JTB255 marine benthic group | 86 | Uncultured bacterium clone F06 | KT336050.1 (99) | Mn nodule | Blöthe,M., et al. 2015 |
| 84 | Alphaproteobacteria | *Rhodospirillaceae*_uncultured | 86 | Uncultured bacterium clone SCB39 | JX227511.1 (99) | Sediment | Wu,Y.-H., et al.2016 |
| 56 | Gammaproteobacteria | JTB255 marine benthic group | 86 | Uncultured bacterium clone F06 | KT336050.1 (99) | Mn nodule | Blöthe,M., et al. 2015 |
| 51 | Alphaproteobacteria | *Rhodospirillaceae*_uncultured | 85 | Uncultured bacterium clone SCB39 | JX227511.1 (99) | Sediment | Wu,Y.-H., et al.2016 |
| 74 | Gammaproteobacteria | JTB255 marine benthic group | 85 | Uncultured bacterium clone F06 | KT336050.1 (99) | Mn nodule | Blöthe,M., et al. 2015 |
| 67 | Gammaproteobacteria | JTB255 marine benthic group | 84 | Uncultured bacterium clone F06 | KT336050.1 (99) | Mn nodule | Blöthe,M., et al. 2015 |
