# Supplementary figures and images for: From the Surface to the Deep-Sea: Bacterial Distributions across Polymetallic Nodule Fields in the Clarion-Clipperton Zone of the Pacific Ocean
Source: Front Microbiol. 2017 Sep 8;8:1696. doi: 10.3389/fmicb.2017.01696 (PMC5596108; doi:10.3389/fmicb.2017.01696)

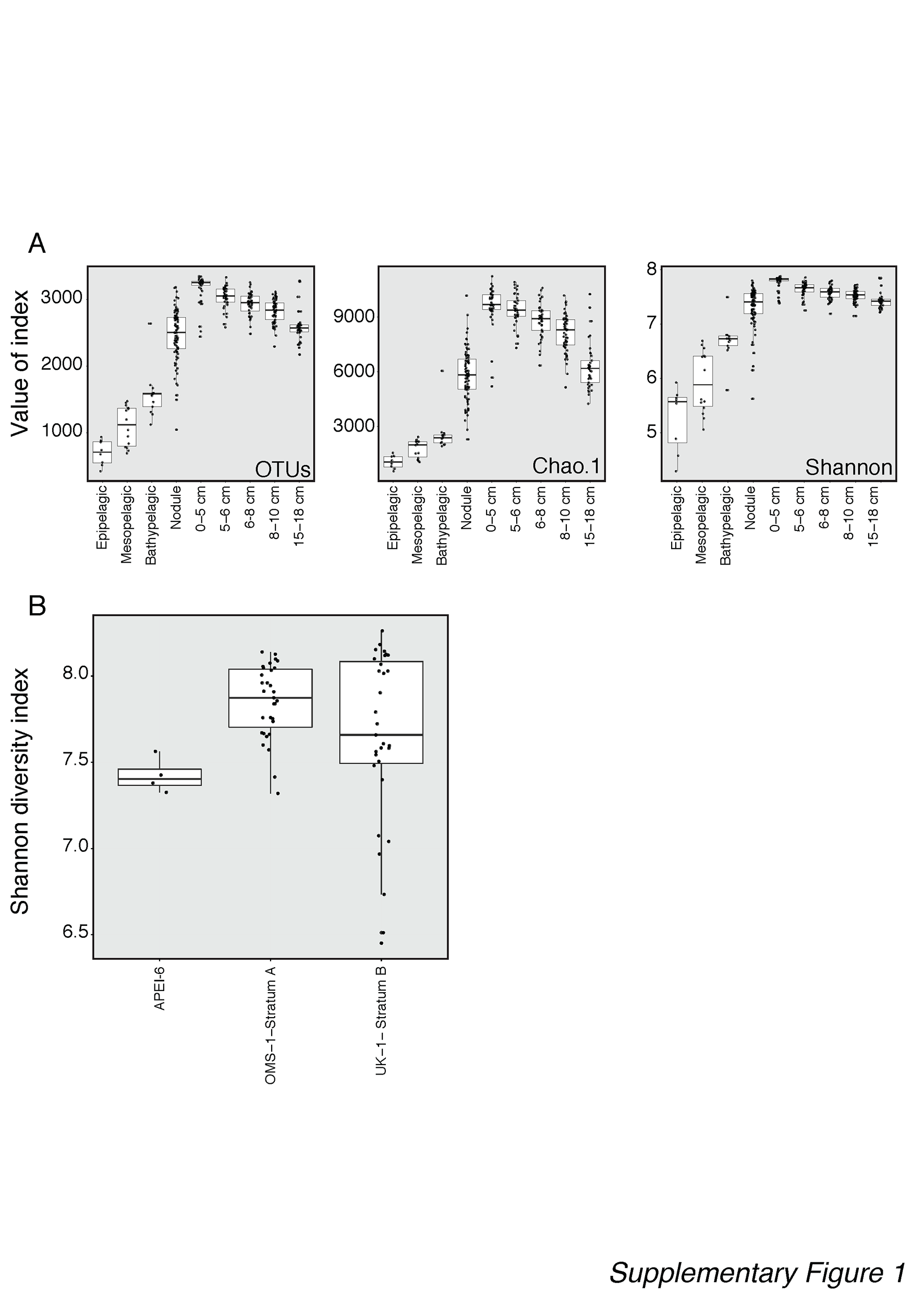

Supplement: Supplementary file 2 [file Image1.TIFF]

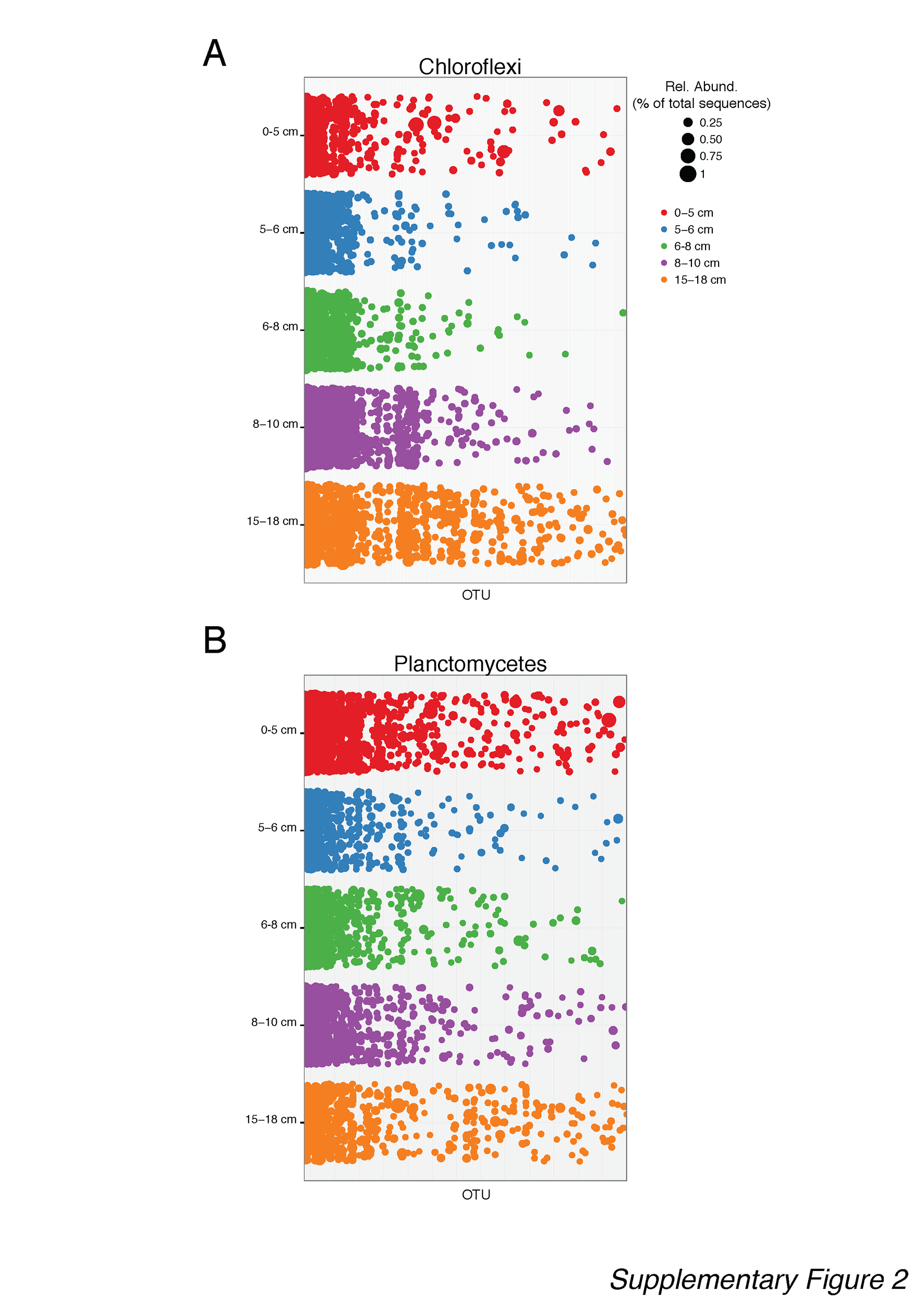

Supplement: Supplementary file 3 [file Image2.TIFF]

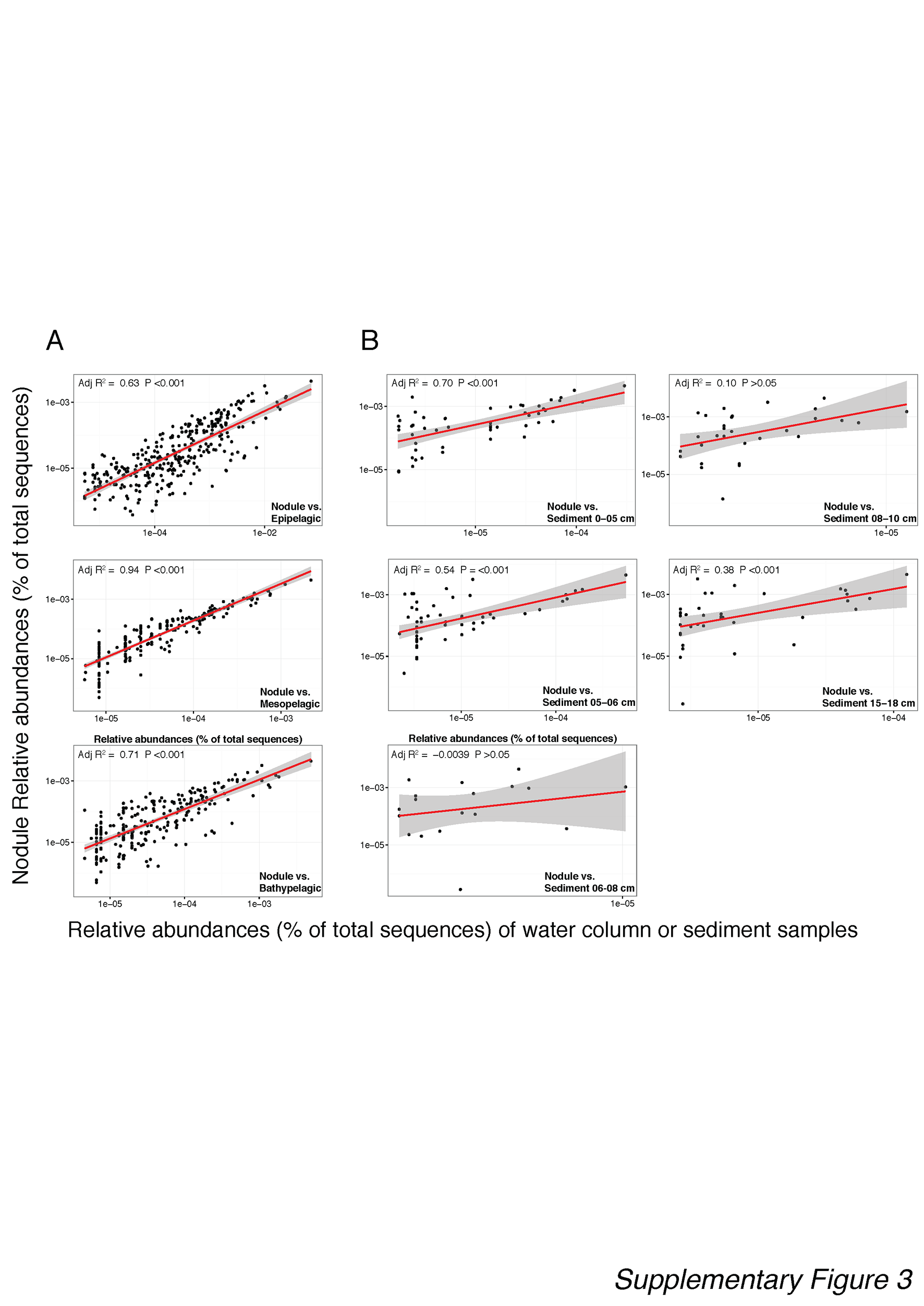

Supplement: Supplementary file 4 [file Image3.TIFF]

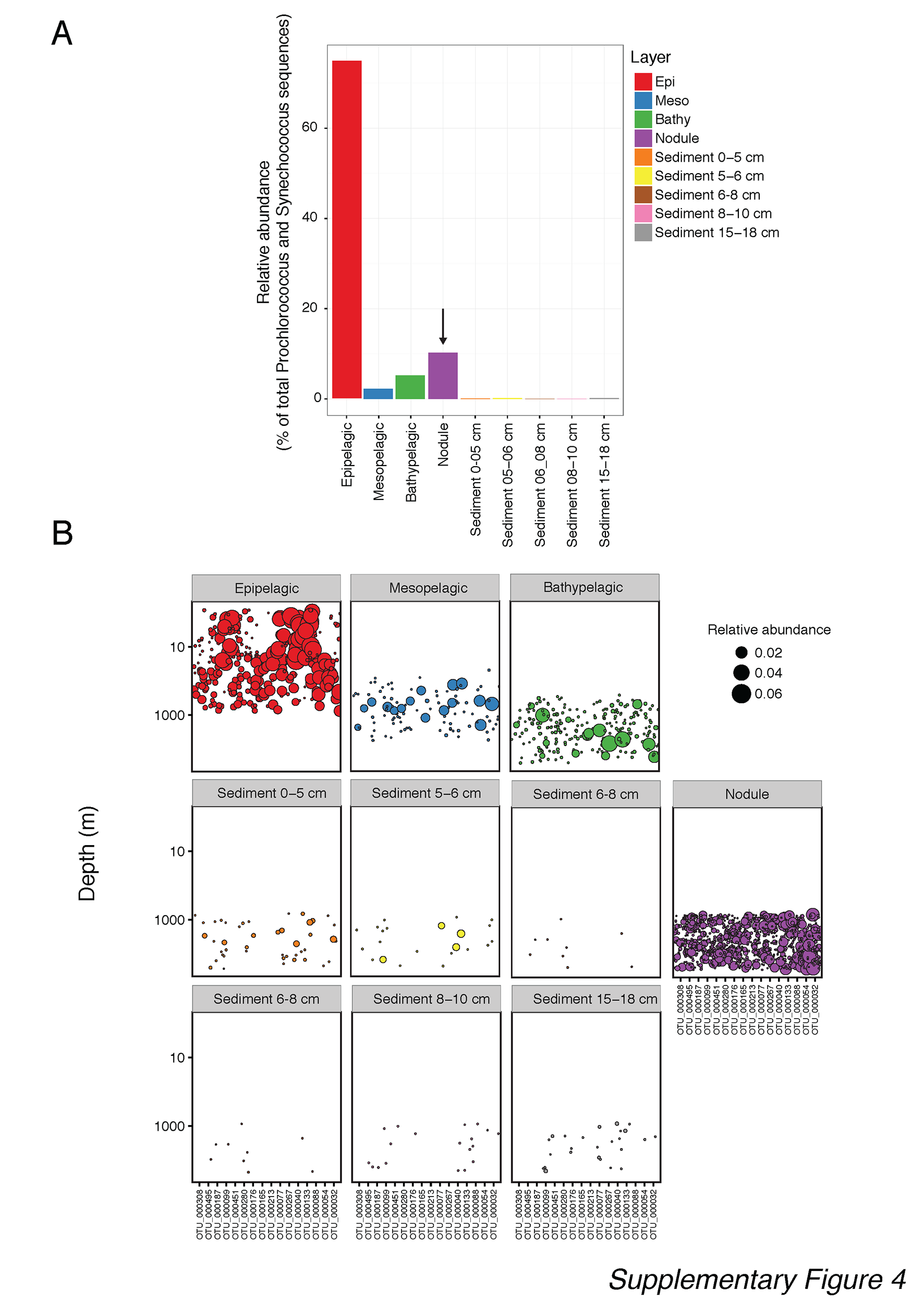

Supplement: Supplementary file 5 [file Image4.TIFF]

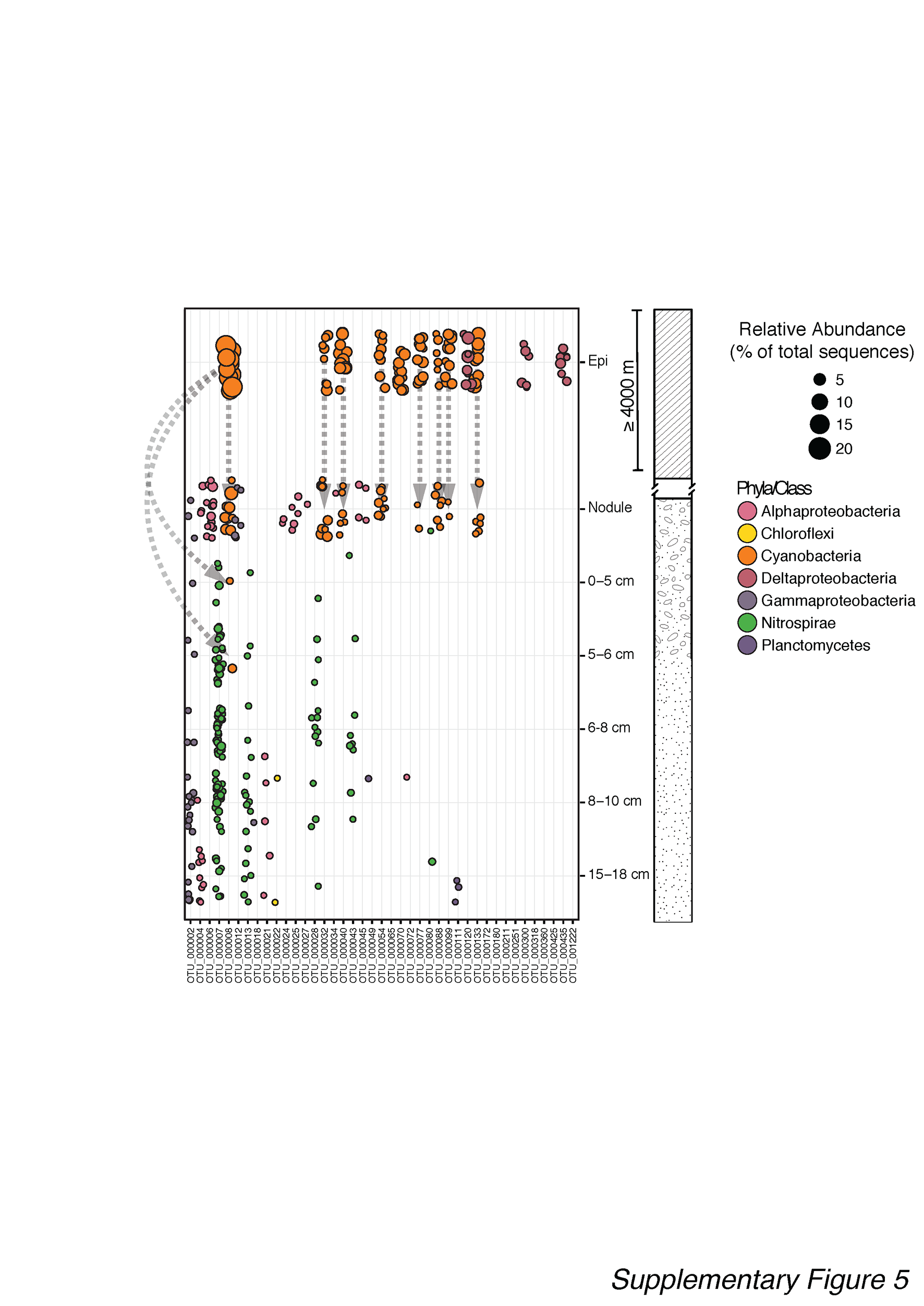

Supplement: Supplementary file 6 [file Image5.TIFF]

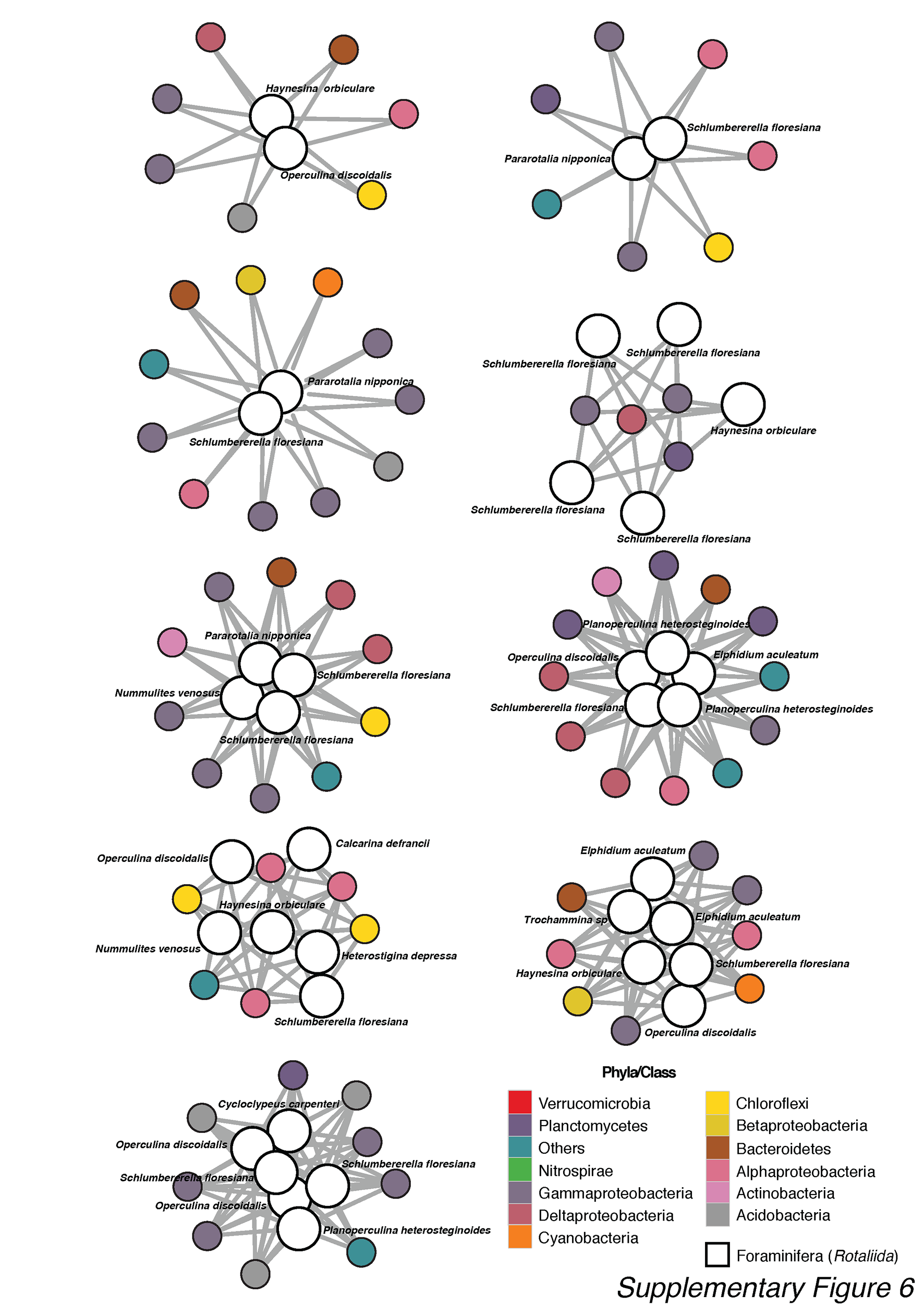

Supplement: Supplementary file 7 [file Image6.TIFF]
